# Assessment of the impacts of climatic variability and anthropogenic stress on hydrologic resilience to warming shifts in Peninsular India

**DOI:** 10.1038/s41598-018-32091-0

**Published:** 2018-09-14

**Authors:** Jhilam Sinha, Ashutosh Sharma, Manas Khan, Manish Kumar Goyal

**Affiliations:** 1 Department of Civil Engineering, Indian Institute of Technology, Guwahati, 781039 India; 2 Discipline of Civil Engineering, Indian Institute of Technology, Indore, 453552 India

## Abstract

Most parts of the world are witnessing climatic warming and the trend is expected to increase in the future. It is important to assess the response of watershed hydrology to this warming. Moreover, human interactions and climatic variability influence the water balance of a catchment. We perform contribution analysis along with resilience study using Budyko framework and two parameters (dynamic deviation and modified elasticity), in-order to comprehend the involvement of anthropogenic stress and climatic variance on partitioning of precipitation and their relation with hydrologic resilience to warming shifts across 55 catchments in peninsular India. Here, 23 catchments have displayed hydrologic resilience (low departure and high elasticity) to climatic warming shifts. Only 37.14% of anthropogenic dominated catchments (higher contribution from human activities in runoff changes) were found to be resilient whereas 58.82% of climate dominated catchments had resilience attributes. Most of the catchments on western and extreme southern part of India were not hydrologic resilient. Extensive human interactions tend to depart the catchment from expected hydrological functioning under critical climatic conditions (Warming in our study) that lead to declining of hydrological resilience.

## Introduction

Global warming has become a topic of immense importance in the field of hydrology for the past few decades^1,2^. Studies have shown an intensification of global water cycle due to warming (includes human induced warming) due to addition of greenhouse gases^3^ that ultimately would lead to more evaporation and severe rainfall events^1,4,5^. Moreover, it interferes with the spatial and temporal patterns of precipitation leading to unequal distribution of it that may again cause precipitation extremes^6^. A significant warming trend of 0.05 °C/10 year in the mean annual temperature over India has been registered during the period 1901–2003^7^. The rate accelerated for the duration (1971–2003) to 0.22 °C/10 year^7^. Moreover, the authors^7^ have documented a trendless behavior of diurnal temperature range during the above mentioned period, due to the recent increase in minimum temperatures.

There has been a global concern on the issue of runoff generation at catchment scale in this changing world^8,9^. Inconsistency in runoff generation due to climate change brings difficulties in its prediction. Any sustainable hydrological response to climate warming would highlight the resilience of that catchment. The concept of resilience was first introduced by Holing^10^ in ecological studies. He emphasized on two different aspects of resilience and contrasted the ideas of stability, consistency with persistency and change that later on was defined as engineering and ecological resilience^11^. Engineering resilience requires a system to remain near to a stable state and resilience is the ability to endure perturbations and come to its stable equilibrium state back. Ecological resilience explains the co-existence of more than one stable state where the system has the tendency to move from one to another and resilience here is measured as the ability to absorb the disturbances before it changes the variables and regime of processes.

The unpredictability nature of hydrological response can create disturbances in planning and establishment of infrastructures for disaster management plans. Creed *et al*.^12^ nicely justified the fusion of resilience with watershed hydrology. Further, Budyko framework was introduced for quantification of resilience. The Budyko framework explains the relationship between precipitation (P), potential evapotranspiration (PET) to discharge and actual evapotranspiration (AET) and provides an idea of partitioning of precipitation to stream flow and AET. It suggests that the portion of precipitation partitioned to runoff, decreases with increase in dryness index (DI; PET/P) which is the ratio of PET to precipitation^13^. Resilience was summarized as the ability of the catchments to maintain this functionality as predicted by the Budyko curve following perturbations^12^. Thus, it is a useful tool to illustrate the influence of climate variability on the hydrology at catchment scale^12,14^. Creed *et al*.^12^ with the help of Budyko framework assessed the influence of forest type and age on streamflow generation under warming conditions from the headwater catchments across North America. Moreover, two indices were introduced to quantify resilience by evaluating the locations of DI (Dryness index) and EI (Evaporative index: ratio of actual evapotranspiration to precipitation; AET/P) points from the Budyko curve: Dynamic deviation (d) and elasticity (e)^12^. Dynamic deviation was described as the extent of departure of catchment’s EI from the Budyko curve following climate warming (cool to warm period). Elasticity was defined as the ratio of range in water year DI values to the difference between the maximum and minimum residual EI values (deviation of EI from Budyko curve). Helman *et al*.^14^ adopted the same methodology to determine the resilience of forested catchments following climatic drought from Eastern Mediterranean section of Israel.

In recent times, along with climate change, human interactions have also disturbed the global water cycle by withdrawing groundwater, surface water to meet the demands and changing land cover^15,16^. It is important to analyze catchment response to anthropogenic stress and climate variability to better understand the watershed hydrology. Wu *et al*.^9^ have adopted Budyko framework and climate elasticity method to quantify the contribution of anthropogenic activities and climatic variability on alteration of seasonal water yield generation on the Loess Plateau, China using eight Budyko based equations (Table 1). Since, India is the second most populated country in the world, it has a huge demand for fresh water which further results in increasing the gap between freshwater demand and supply^17^. India’s booming economy in recent years have accelerated urbanization and industrialization and this led to changes in Land Use and Land Cover (LULC) in most parts of the country^18^. For instance, the city Hyderabad in Telangana state has shown a tremendous LULC change over last four decades^19^. The Hasdeo river basin in the northern part of Chhattisgarh state has also undergone a tremendous LULC change from degradation of dense forest (−7.52%) to increase in non-forest (+8.59%) and open forest land (+3.55%) change^20^. Moreover, urbanization was the major driver of land conversions in India after the 1950s due to accelerated population growth rate^21^. In fact, during 2001–2011, urbanisation increased faster with urban population growth more than rural population increment for the first time since independence^22^.

**Table 1:** The eight Budyko-type equations (including both parametric and non-parametric equations) considered in the study. ‘NP’ denotes the non-parametric type equations.

Equation names	Equations	Parameters	References
BDK-S	$\frac{E}{P}=1-{e}^{\frac{-{E}_{0}}{P}}$	NP	Schreiber^58^
BDK-O	$\frac{E}{P}=\frac{{E}_{0}}{P}tanh(\frac{P}{{E}_{0}})$	NP	Ol’dekop^59^
BDK	$\frac{E}{P}=\sqrt{\frac{{E}_{0}}{P}tanh(\frac{P}{{E}_{0}})(1-{e}^{\frac{-{E}_{0}}{P}})}$	NP	Budyko^60^
BDK-PT	$\frac{E}{P}=\frac{1}{\sqrt{1+{(\frac{{E}_{0}}{P})}^{-2}}}$	NP	Pike^61^ Turc^62^
BDK-FY	$\frac{E}{P}=1+\frac{{E}_{0}}{P}-{(1+{(\frac{{E}_{0}}{P})}^{\omega })}^{\frac{1}{\omega }}$	ω	Fu *et al*.^36^, Yang *et al*.^32^
BDK-Z	$\frac{E}{P}=\frac{1+w\frac{{E}_{0}}{P}}{1+w\frac{{E}_{0}}{P}+{(\frac{{E}_{0}}{P})}^{-1}}$	w	Zhang *et al*.^29^
BDK-CY	$\frac{E}{P}={(1+{(\frac{{E}_{0}}{P})}^{-n})}^{\frac{-1}{n}}$	n	Choudhury^51^, Yang *et al*.^31^
BDK-WT	$\frac{E}{P}=\frac{1+\frac{{E}_{0}}{P}-\sqrt{{(1+\frac{{E}_{0}}{P})}^{2}-4\varepsilon (2-\varepsilon )\frac{{E}_{0}}{P}}}{2\varepsilon (2-\varepsilon )}$	ε	Wang and Tang^30^

Therefore, human interactions will affect the partitioning of precipitation at the catchment scale. Apparently, it would not be erroneous to say that anthropogenic activities would have a good hand in controlling the resilience of a catchment under climate warming. To answer the argument, a plausible attempt has been taken in the present study, to combine the contribution analysis with resilience concept for investigating the partitioning of precipitation to ET and streamflow under warming condition and to assess the influence of anthropogenic activities on resilience. Using the two metrics presented by Creed *et al*.^12^ of which, the elasticity metric has been modified in our study (Modified elasticity: e_m_; Discussed in method section) and the 8 budyko based equations (Table 1), the resilience of catchments to climatic warming is studied. Note that, the climate elasticity method in contribution analysis should not be confused with the modified elasticity index used in resilience study due to similarity in name. In this study, 55 catchments have been taken into consideration from 17 river basins across peninsular India, defined according to the report “Watershed Atlas of India” of India-WRIS (Water Resources Information System) are selected^23^. Due to data unavailability, 3 basins could not be taken- Ganga Basin, Brahmaputra Basin, and Indus Basin. The catchments encompass about 13.25% of the country’s land mass as shown in Supplementary Fig. S1. Details of all the meteorological parameters for each of the basins are given in Supplementary Table S1. The purpose of this study is to: (1) To analyze the quantitative contribution of human activities and climatic variability during the period of 1988–2011, implementing climate elasticity method based on eight Budyko based theories, (2) To discuss the attribution diverseness among the catchments, (3) To study resilience of the catchments and (4) To analyze the influence of anthropogenic and climatic variability on the resilience of the catchments.

## Results

### Trends Analysis of Long-Term Hydro-Meteorological Variables

The existing trends in P, ET_0_ and Q are shown in Supplementary Table S2 with Z statistics, significance of Non-parametric Mann Kendall test^24,25^ and magnitude of the trend (slope) by Sen’s slope estimator test^26,27^ for 55 catchments during the period of 24 years (1988–2011). Almost all the catchments of river basins like Baitarni and Brahmani basin (ID 1–4), Godavari (ID 16, 17, 19–24), Krishna (ID 25–27), Mahi (ID 34), Narmada (ID 36–38), Sabarmati (ID 41), and Tapi (ID 44) are facing the challenges of downward trend of runoff generation. The annual runoff of many catchments have undergone downward trend but their annual precipitations have shown an increasing rate, indicating that precipitation is not the sole reason for changes in the long-term runoff generation. The trends have been discussed in the supplementary information.

### Change Point Analysis of Basin and Climatic Characteristics

Determination of baseline period for the catchments is done, considering significant change points in time series of ω (Basin parameter in BDK-FY), P, PET and Q. Pettitt test^28^ is applied to the time series of ω, P, PET and Q to check their non-stationarity. Information on time series is given in supplementary information. From the change point analysis, in case of ω, significant change points are mainly found in between 1996 and 2005 for 39 catchments out of 55 (35 catchments at 95% and 4 catchments at 90% significance level). In case of P, 7 catchments show significant change points which vary from 2003 to 2004 with 24 catchments for PET showing significant change points from 1995 to 2004 whereas for Q, 6 catchments show significant change points in between 1995 and 2005 (Supplementary Table S3). Overall, change points of ω, P, Q and PET lie mainly in between 1995 and 2005. Moreover, urban population growth was higher than rural population growth for the first time in India since independence during 2001–2011^22^. Therefore, a common baseline period from 1988 to 1997 has been adopted while 1998–2011 is chosen for the assessment period. Following the analysis, within the two sub-periods (i.e. 1988–1997 and 1998–2011), ω is remaining almost stationary.

### Aridity Index and Runoff Elasticity Coefficients

**Figure 1:**
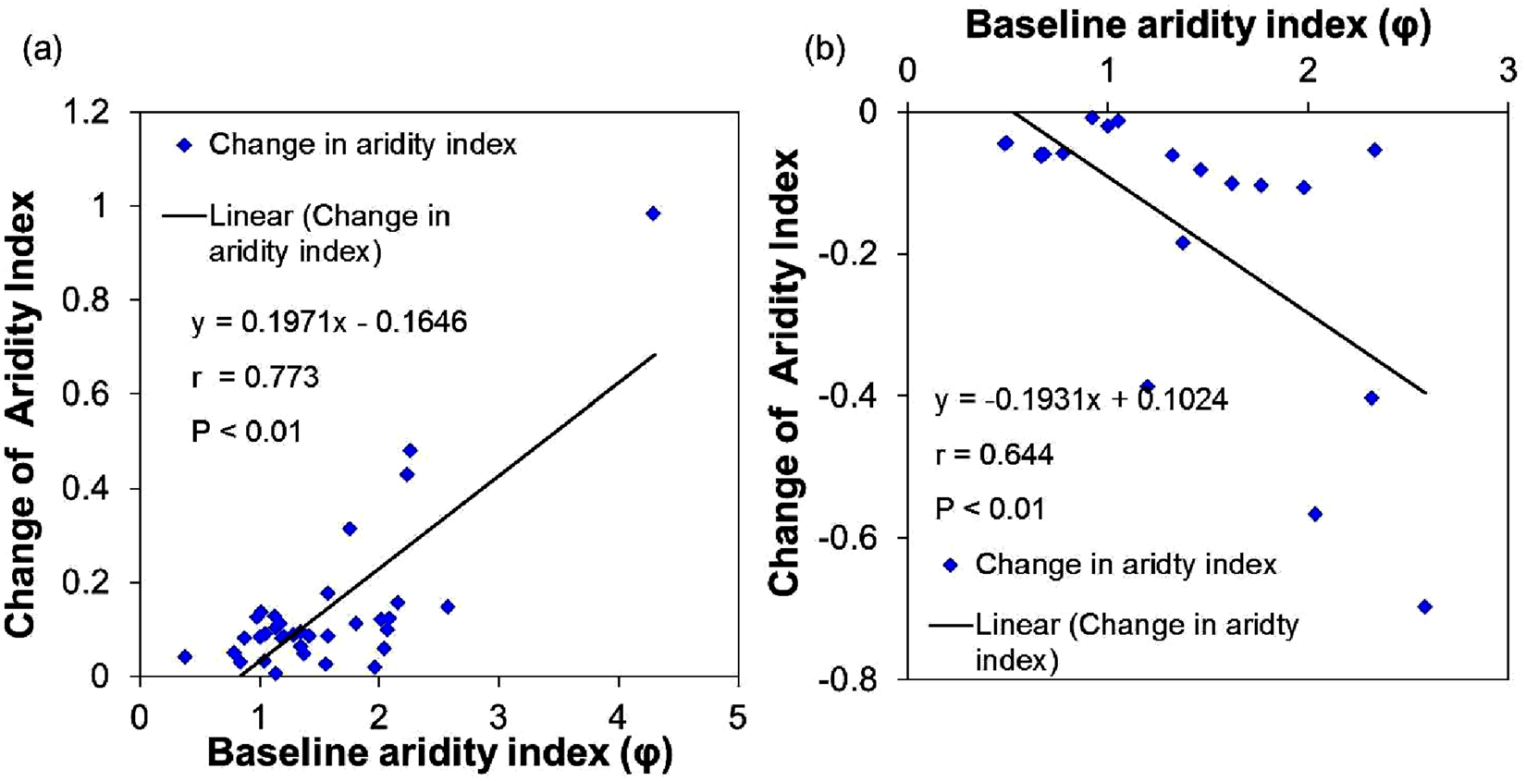
Relationship between change in aridity index from baseline to assessment period and aridity index during baseline period. (**a**) Shown for the catchments that undergone positive change. The trend line is modelled for y = 0.197 × −0.165. (**b**) Shown for the catchments that undergone negative change. The trend line is modelled for y = −0.193 × +0.102.

Maximum aridity index in both the periods is found for the catchments in the northwestern part of India (Supplementary Fig. S2a). During the baseline period, the aridity index ranged from 0.39 to 4.30 for Erinzipuzha (ID 49) and Gandhav (ID 45) catchments respectively with a mean value of 1.47 (Supplementary Table S4). This mean value has increased to 1.50 for the assessment period and ranged from 0.43 to 5.29 again for Erinzipuzha and Gandhav catchments respectively. Despite an increment, 20 catchments from south, extreme west and some part of the east show decrement in aridity index. The catchments in northwestern, central and most part of the east have shown positive change indicating drying of the catchments (Supplementary Fig. S2b). In addition, Fig. 1a,b showed significant positive (R = 0.773, p < 0.01) and negative correlation (R = 0.644, p < 0.01) respectively, between changes in aridity index and baseline aridity index, indicating that catchments with higher aridity index values exhibited a greater change explaining that drier catchments are more liable to change in either direction.

The values of ${{\epsilon }}_{P}$ are more than absolute values of ${{\epsilon }}_{{E}_{0}}$ for all the basins, thus indicating more sensitiveness of runoff changes to precipitation variability than variability in potential evapotranspiration (Supplementary Table S5). Supplementary Fig. S2c,d show the spatial distribution of ${{\epsilon }}_{P}$ and $\,{{\epsilon }}_{{E}_{0}}$ across the catchments respectively.

### Quantitative Attribution of Runoff Change

**Figure 2:**
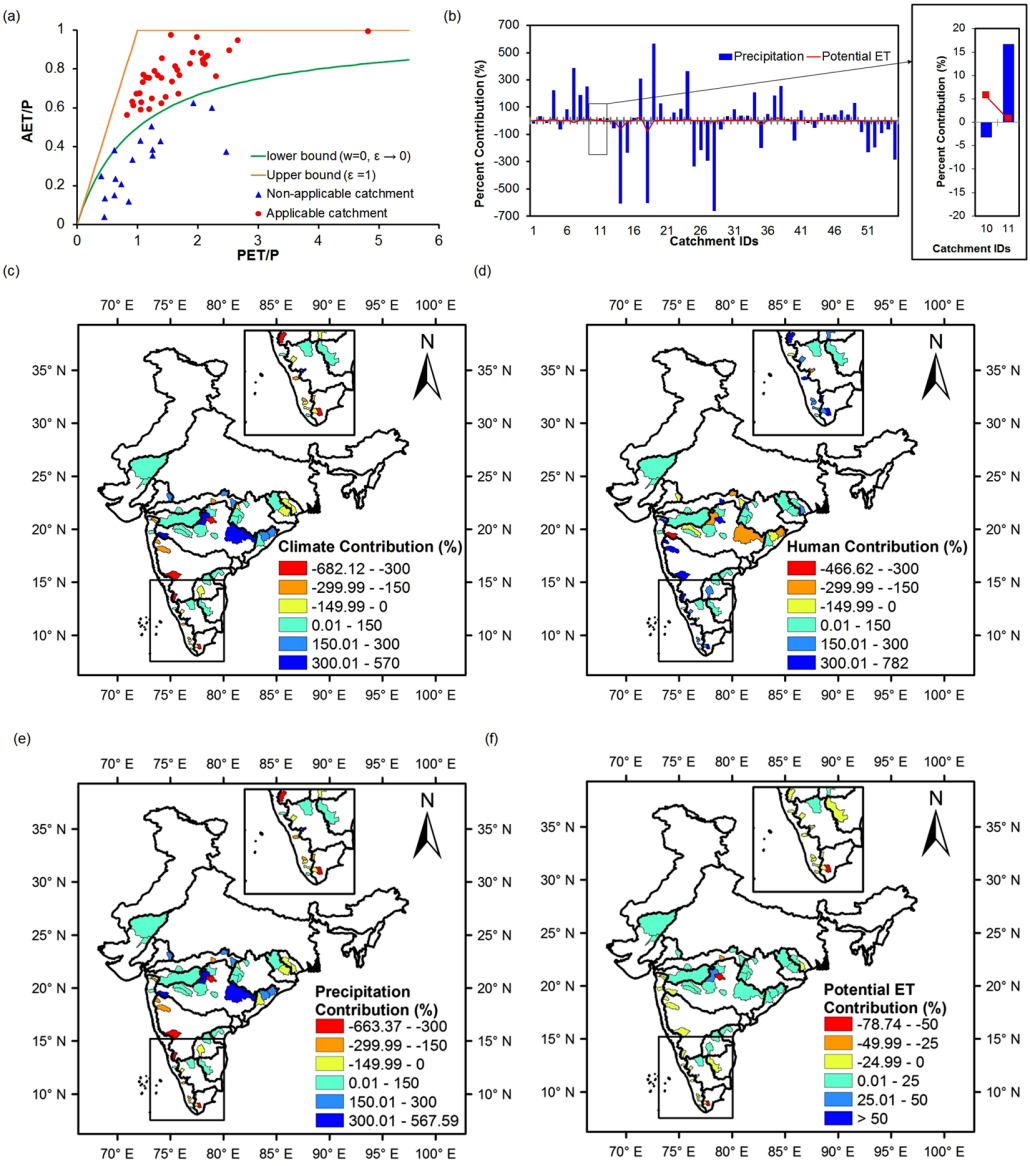
Contribution attributes of the catchments and spatial distribution of percentage contributions. (**a**) Non-applicability of Zhang and Wang and Tang budyko equations on 17 catchments (blue triangles). Lower bounds of the parameters in these two equations are also shown. (**b**) Mean percentage contributions (%) of precipitation and Potential evapotranspiration for the catchments. The percentage contributions of catchment ID 10 is shown separately on the right. Spatial distribution of contributions from (**c**) climatic variability (P and ET), (**d**) human activities, (**e**) variability in precipitation and (**f**) potential evapotranspiration variations across the catchments.

Supplementary Table S6 shows the relative contribution of climate variability to change in runoff from eight Budyko based methods. All catchments except 10 of them (IDs: 6, 18, 19, 35, 39, 40, 43, 48, 53, 55) have experienced a decrement in runoff generation from baseline to assessment period. Anthropogenic activities have greater contributions in 35 catchments (Supplementary Table S6**)**. Negative values of contributions mean the direction of the runoff change (impact) due to climatic variance is opposite to the direction of change of total runoff from baseline to assessment period. If total runoff decreased (increased), then contributing negatively means climatic variance has increased (decreased) the runoff. Out of the 8 Budyko based equation, 2 of them given by Zhang^29^ and Wang and Tang^30^ could not be applied to 17 catchments (Shown as ‘NA’ in Supplementary Table S6) because of the values of the basin parameters in both the equations, coming out of their lower theoretical range (negative values in both the cases). It is seen from Fig. 2a that the evaporative index (AET/P) for these 17 catchments is lesser than the suggested values by the lower bound of budyko curves. A possible explanation might be that excess water from ground-flow due to irrigation is contributing to these 17 catchments, consideration of which has reduced the actual ET according to water balance equation. Moreover, Yang *et al*.^31^ demonstrated that Zhang’s equation does not follow wet boundary condition (Energy limit) indicating that the equation is not an analytical solution to the long-term water-energy balance equation, that raises questions on its applicability. Even, Wang and Tang^30^ stated that watershed data were found below the lower bound (ε < 0; negative value), meaning out of range for a few studies^32,33^ similar to what we found.

The results obtained from eight Budyko-based methods for each catchment are not consistent (but consistent with sign for each catchment), likely due to different methodologies of considering the hydro-meteorological parameters and the inclusion of basin characteristics to explain the budyko framework. Despite the inconsistency, averaging the percentage contributions does project the impact of climatic variance on runoff changes. It can be seen that for climatic variability, most of the catchments in the central, western and all in northwestern part show positive mean contributions with maximum value of 566.62% for Pachegaon (ID 19) whereas most of the catchments in the southern, extreme western and extreme eastern part show negative contributions with Nandgaon having the highest contribution of −682.12% (ID 18) (Fig. 2c). Spatial variation of mean contribution from anthropogenic stress showed similar trends as that of climatic variability (but with opposite extremes), since percentage contributions from anthropogenic stress and climate variability equals 100 (Fig. 2d). Also, all catchments except only 12 have contributed positively. This indicates that human activities in most of the catchments have caused runoff change in the same direction, annual average runoff has actually changed.

One of the key intentions of the study is to observe the dominant parameter, having highest influence (contribution) on catchment hydrology. A two parameter model is executed to predict climatic influence using P and E_0_ elasticities under climate elasticity method. Figure 2b shows the mean contribution (%) of precipitation and potential evapotranspiration variabilities, in changing of runoff for all the catchments. Further decomposition of evapotranspiration elasticity into more parameter elasticities (wind speed, sunshine duration, relative humidity, maximum and minimum temperature; multi parameter model)^34,35^ is promising (Supplementary Table S7; evapotranspiration elasticities have not been included in the study) for quantifying individual impacts on runoff. However, estimation errors in runoff change due to climatic variance may be propagated due to integrated climatic parameters, which is larger than considering only E_0_ elasticity and thus magnifies uncertainties in estimated outcomes^34^. Therefore, it is advised to implement two parameter model when assessing the effects of climatic variability, where climatic influence is considered as a whole. Multi parameter model is appropriate during evaluation of discrete impacts of climatic variables for management activities^34^. The study emphasizes on climatic influence as one, to determine its dominance on catchment hydrology. The percentage contributions of precipitation variability are greater in all the catchments except Srikakulam (ID 10) which is shown separtely. The catchments in the extreme west, south, east and a few in the center have shown negative contributions of precipitation with a maximum value of −663.37% for Shimoga (ID 28) and highest positive contribution of 567.60% for Pachegaon (ID 19) (Fig. 2e). Catchments from the northwest, south, east and west showed positive contribution of potential evapotranspiration with the highest value of 28.28% for Hivra (ID 17). The highest negative contribution is −78.74% for Nandgaon (ID 18) from the south (Fig. 2f).

### Deviation and Modified Elasticity

The two components of deviation (s, d) along with modified elasticity (e_m_) for each catchment are shown in Table 2. Static deviation ranged from −0.25 for Kheroj (ID 41) to 0.11 for Mahuwa (ID 53). Out of 55 catchments, 39 catchments have shown negative static deviation, which indicates higher water yield generation than the expected value due to inherent properties of the catchments. A positive value indicates lower pre-warming water yields than the expected value. In contrast, 32 catchments were very close to the theoretical curve (|s| < 0.05) indicating water yield similar to predictive values from the theoretical Budyko curve.

**Table 2:** Catchment 3 year cool period (minimum average temperature), 3 year warm period (maximum average temperature), change in temperature during warming shifts, domination in the catchements (anthropogenic and climatic) and resilience indices (s - static deviation, d - dynamic deviation and e_m_- modified elasticity).

ID	Cool Period	Warm Period	ΔT(°C)	s	d	e_m_	Domination
1	1992–1994	2008–2010	0.907	−0.056	0.141	0.006	A
2*	1993–1995	2008–2010	0.925	0.047	0.003	96.510	A
3	1990–1992	2008–2010	0.939	−0.021	0.104	0.086	A
4*	1989–1991	2008–2010	0.881	0.080	0.013	7.170	C
5	1992–1994	2001–2003	0.448	−0.140	0.172	2.208	A
6*	1994–1996	2001–2003	0.528	−0.024	−0.010	36.954	C
7*	1994–1996	2002–2004	0.464	0.044	−0.096	7.143	C
8*	1993–1995	2008–2010	0.856	0.030	−0.016	2.670	C
9*	1993–1995	2008–2010	0.961	0.034	−0.016	4.161	C
10*	1993–1995	2008–2010	0.777	0.012	−0.044	2.202	A
11*	1989–1991	2001–2003	0.624	−0.003	0.061	11.530	A
12*	1992–1994	2001–2003	0.586	0.003	−0.012	102.507	A
13*	1992–1994	2009–2011	0.505	−0.005	0.099	4.242	A
14	1992–1994	2009–2011	0.509	−0.124	0.353	1.427	A
15	1992–1994	2009–2011	0.495	−0.138	0.474	0.836	A
16	1989–1991	2008–2010	0.939	−0.072	0.217	0.657	A
17	1989–1991	2008–2010	0.942	−0.012	0.027	0.353	C
18*	1989–1991	2008–2010	0.96	−0.026	−0.018	4.281	A
19*	1989–1991	2009–2011	0.909	−0.036	0.041	5.499	C
20	1989–1991	2008–2010	0.944	−0.030	0.117	2.307	C
21	1989–1991	2008–2010	0.949	−0.006	0.107	1.731	A
22*	1989–1991	2008–2010	0.976	−0.006	0.050	7.250	C
23	1989–1991	2008–2010	0.99	−0.087	0.134	2.496	C
24*	1993–1995	2008–2010	0.666	0.004	0.000	829.686	C
25*	1992–1994	2001–2003	0.577	−0.045	0.030	32.940	A
26	1989–1991	2009–2011	0.885	−0.220	0.441	0.390	A
27*	1989–1991	2002–2004	0.829	−0.110	0.095	7.563	A
28*	1990–1992	1998–2000	0.471	−0.080	−0.035	1.726	A
29	1990–1992	2008–2010	0.755	−0.094	0.151	0.309	A
30	1989–1991	2008–2010	0.714	−0.095	0.189	0.395	A
31*	1990–1992	2008–2010	0.763	−0.012	−0.023	13.666	C
32	1990–1992	2008–2010	0.799	−0.044	0.137	1.581	A
33*	1990–1992	2008–2010	0.832	−0.030	0.068	3.698	A
34	1995–1997	2009–2011	0.737	−0.001	−0.106	4.249	C
35	1989–1991	2008–2010	0.908	0.013	−0.101	1.466	A
36	1989–1991	2008–2010	0.75	−0.206	0.333	0.613	N
37	1989–1991	2008–2010	0.809	−0.010	0.123	2.484	C
38*	1989–1991	2009–2011	0.988	−0.009	−0.057	2.228	C
39*	1989–1991	2001–2003	0.657	0.043	0.017	9.497	A
40*	1992–1994	2001–2003	0.623	0.036	−0.056	2.209	A
41	1992–1994	2009–2011	1.206	−0.252	0.304	1.474	N
42	1990–1992	2008–2010	0.858	−0.037	0.077	0.441	A
43	1992–1994	2008–2010	0.875	0.090	−0.179	0.495	A
44	1989–1991	2008–2010	0.851	−0.065	0.087	0.913	C
45*	1992–1994	2009–2011	1.314	0.000	0.009	82.467	A
46	1992–1994	2009–2011	1.207	−0.143	0.201	3.323	A
47	1991–1993	2009–2011	0.461	−0.085	0.240	0.216	N
48	1989–1991	2009–2011	0.826	0.038	−0.168	0.016	A
49	1990–1992	1998–2000	0.506	−0.063	0.163	0.057	C
50	1992–1994	2009–2011	0.475	−0.040	0.065	0.559	A
51	1992–1994	2002–2004	0.411	−0.222	0.417	0.233	A
52	1992–1994	2009–2011	0.468	−0.034	0.098	0.088	A
53	1989–1991	2009–2011	0.761	0.105	0.080	0.525	A
54	1992–1994	2009–2011	0.492	−0.115	0.293	0.019	A
55	1989–1991	2009–2011	0.806	0.083	−0.253	0.279	A

A, C, N indicate anthropogenic activities, climatic variability and neutral (both) respectively as primary contributor to changes in runoff. *Indicates that the catchment is resilient to climatic warming.

Dynamic deviation showed a wide range of value chiefly above the theoretical curve. It ranged from −0.25 for Gadat (ID 55) to 0.47 for Theni (ID 15). Only 17 catchments have shown negative d value indicating higher than expected water yield during warming conditions. We used a threshold value of 0.1 for dynamic deviation (d) to differentiate a catchment, resistant to climatic variance (warming in our case) from non-resistant catchments. In total, 29 catchments showed |d| < 0.1, but not all are resilient. A catchment is resilient when its response to changes in climate, propagates along the curve with marginal departures from it (low departure, high elasticity; Supplementary Fig. S3).

Modified elasticity ranged from 0.01 for Anandapur (ID 1) to 829.69 for Pathagudem (ID 24) (Table 2). Some of the catchments showed a very high value of modified elasticity. A possible reason may be because the theoretical Budyko curve was produced with the parameter ‘ω’ representing catchment soil, topographical properties for each of the catchment^36^ calculated during contribution analysis. Twenty-one catchments have shown e_m_ < 1 signifying narrower range in DI_A_ than EI_R, A_. Rest of the catchments showed broader range in DI_A_ resulting in high modified elasticity.

### Budyko Metrics with warming

**Figure 3:**
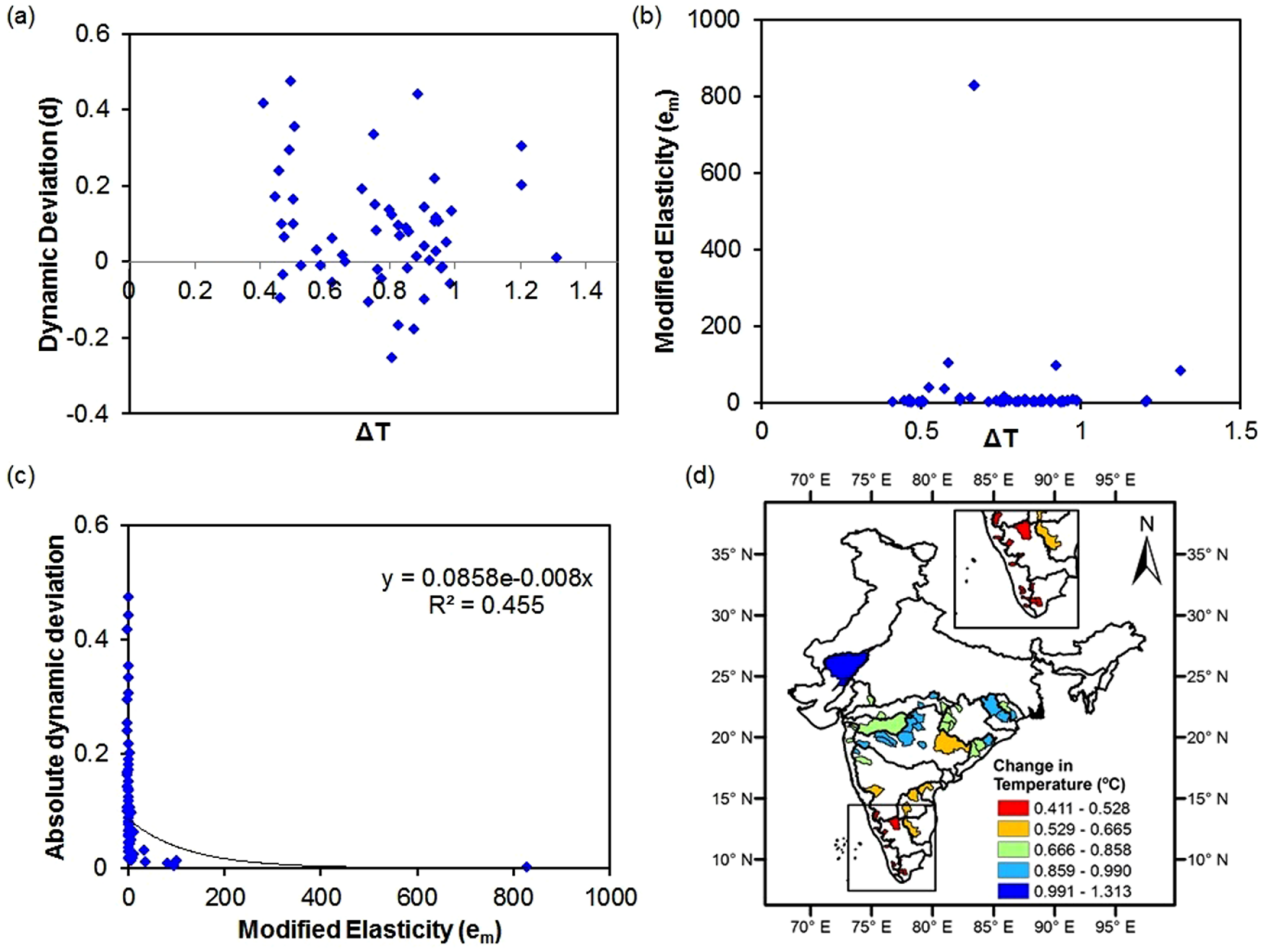
Attribution of Resilience indices and extent of temperature change. Relationship of (**a**) deviation and (**b**) modified elasticity with ΔT (Difference in temperature between cool and warm period). (**c**) Relationship between absolute dynamic deviation and modified elasticity for all the catchments. The trend line is modelled with y = 0.086e^−0.008x^; (**d**) Spatial distribution of difference in temperature (∆T) from cool to warm period.

There is no concrete relationship of dynamic deviation and modified elasticity with temperature differences (Fig. 3a,b**)**. Wetter conditions due to heavy precipitation may have diminished the effect of warming in these catchments^12^. Further, ∆T is observed to be maximum in the north western, western and some part of eastern India. However, lowest difference has been observed in extreme southern part of India (Fig. 3d**)**.

Dynamic deviation varied widely with modified elasticity (not shown) but a fair relationship appeared when we took the absolute value of ‘d’ (Fig. 3c). An exponential curve has justified the relationship indicating lower deviation with an increase in modified elasticity (R^2^ = 0.455). Further, the relationship remained the same when the catchments were divided into two groups based on their ΔT (not shown).

## Discussion

**Figure 4:**
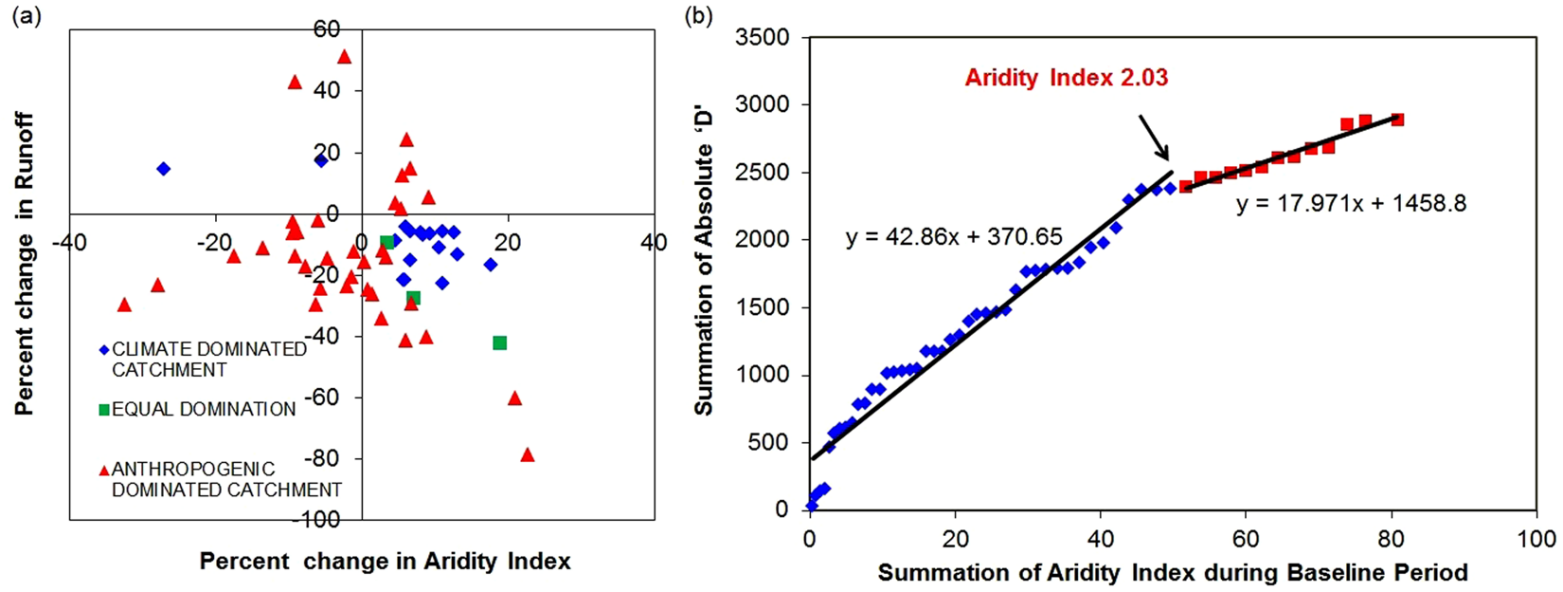
Influence of climatic and anthropogenic stressors. (**a**) Heterogeneity among the catchments in context to percentage change in runoff and aridity index from baseline to assessment period, among the catchments. (**b**) Relationship between summation of absolute ‘D’ and summation of aridity index during baseline period (1988–1997).

Our key objective was to recognize the influence of climatic variance and anthropogenic stress in partitioning of precipitation (Fig. 4). The catchments are separated into 4 groups based on their location in the quadrants (Fig. 4a**)**. Quadrant 1 and 4 represent the condition where catchments have become drier with quadrant 2 and 3 showing wetter conditions in assessment period. Budyko hypothesis suggests that with increase in aridity index, water availability decreases. Evidently, contributions from climatic variance should behave according to budyko curve, i: e climatic variance should decrease runoff in case of quadrant 1 and 4 whereas it should increase runoff in catchments from quadrant 2 and 3. It is seen (Supplementary Tables S4 and S6) that climatic variance has contributed negatively in all catchments in quadrant 1 (ID 18, 35, 40, 43, 53, 55) and 3 (ID 1, 3, 5, 13–15, 25–29, 42, 50–52, 54) because climatic variance has decreased runoff in catchments of quadrant 1 and increased runoff in quadrant 3 (opposite in direction to where total runoff has actually changed and thus negative contribution). Similarly, it contributed positively in all catchments from quadrants 2 (ID 39, 48, 6, 19) and 4 (ID 2, 4, 7–12, 16, 17, 20, 21–24, 30–34, 37, 38, 44–46, 49) because climatic variance has increased runoff in quadrant 2 and decreased it in quadrant 4 (same in direction to where runoff has actually changed and thus positive contribution). Climatic variance is the primary contributor (higher contribution from climatic variability) in climate dominated catchments. Also, it is important to note that, the primary contributor will contribute in the same direction, runoff actually changes (positive contribution). Figure 4a clearly shows that all catchments in quadrant 1 and 3 are dominated by anthropogenic activities (higher contribution from anthropogenic stress; Supplementary Table S6) indicating the fact that due to anthropogenic stress (Deforestation, urbanisation and withdrawals), the hydrological system functioned differently to what suggested by the Budyko curve. In quadrant 1, six anthropogenic dominated catchments showed an increase in runoff with highest increment in Govindpur (ID 43), that might be due to urbanization and deforestation of the catchments. Decrement in runoffs for anthropogenic dominated catchments in quadrant 3 with highest decrement in A.P Puram (ID 13) is the clear indication of high intensity of withdrawals or water conservation measures in these catchments. Moreover, human activities in anthropogenic dominated catchments have introduced additional increment (quadrant 2) and decrement (quadrant 4) in runoff change with highest increase in Alladupalli (ID 39, quadrant 2) and highest decrement in Gandhav (ID 45, quadrant 4, p < 0.01 for annual discharge in Supplementary Table S2) respectively. These imparities among the catchments decide the required conservational measures to be taken for maintaining ecological balance at the catchment scale.

**Figure 5:**
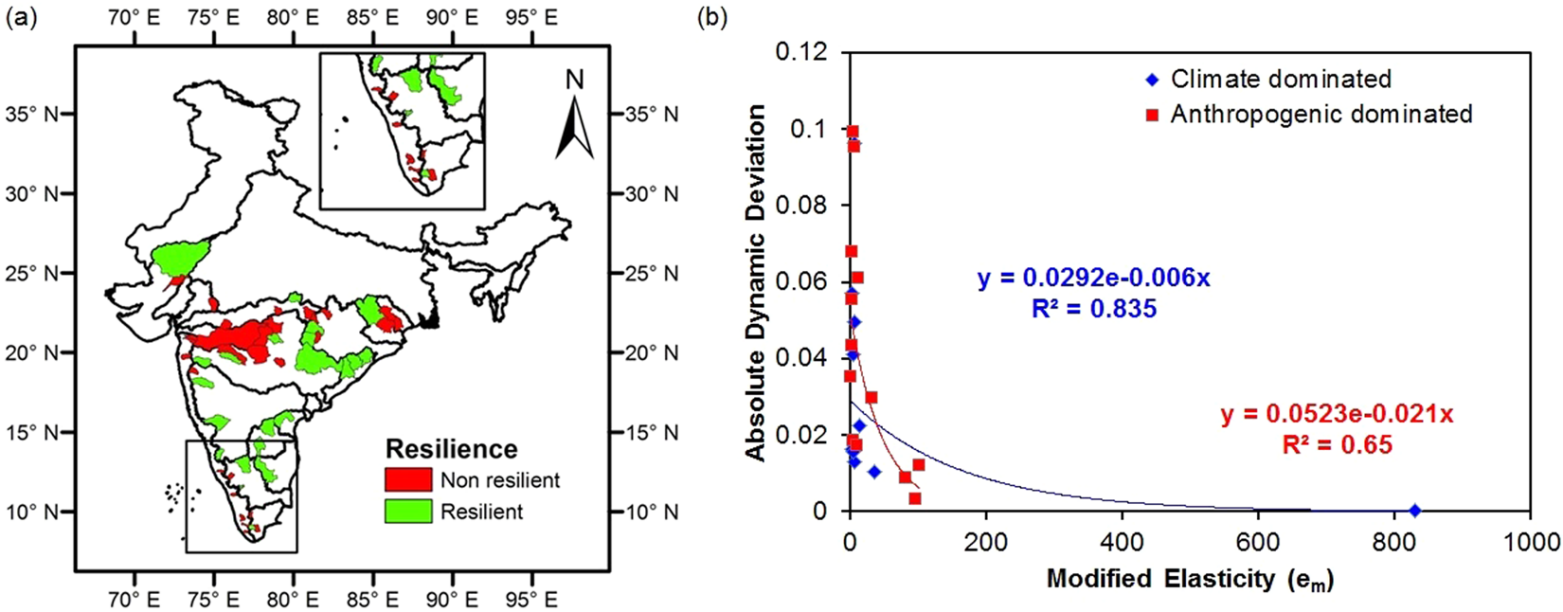
Resilience of catchments and relationship of resilience indices for resilient catchments. Spatial distribution of (**a**) the resilient catchments, where twenty-three catchments have shown resilience attributes among 55 catchments and (**b**) relationship between absolute dynamic deviation and modified elasticity for resilient catchments. The trend line for climate dominated resilient catchments (shown with blue line) is modelled with y = 0.029e^−0.006x^. The trend line for anthropogenic dominated resilient catchments (shown with red line) is modelled with y = 0.052e^−0.021x^.

Out of 55 catchments, 23 catchments have exhibited resilience to climatic warming shifts (low dynamic deviation, |d| < 0.1 and high modified elasticity, e_m_ > 1) as shown in Table 2. Most of the catchments from the eastern part and upper southern part of India are resilient whereas, most of the catchments in the western part are non-resilient (Fig. 5a). Table 2 also shows the dominant variable in changing of partitioning of precipitation to runoff and evapotranspiration. Thirteen resilient catchments are anthropogenic dominated whereas 10 resilient catchments are climatic dominated. This highlights the fact that only 37.14% (13/35 catchments) of the anthropogenic dominated catchments are resilient whereas 58.82% (10/17 catchments) of climate dominated catchments have shown resilience characteristics. It therefore, signifies the negative impact of interaction of human activities on the resilience of catchments. This may have resulted due to extensive human interactions that tend to drag the response away from the theoretical curve. This would broaden the EI range and thus, could decrease the modified elasticity. Figure 5b shows that anthropogenic activities have led to more dynamic deviation than climatic variance for the same elasticity values upto around e_m_ = 40, covering most of the catchments. The relationship between absolute dynamic deviation and modified elasticity (exponential) is stronger for catchments that are dominated with climatic variability (Climatic variance: R^2^ = 0.835, Anthropogenic stress: R^2^ = 0.650). Further, weak linear negatively correlated relationship (decrease in modified elasticity with increase in contribution; higher interaction lowering modified elasticity) was obtained between percentage anthropogenic contributions and modified elasticity (R = −0.226, p > 0.05; not shown) along with weak linear positively correlated relationship (increase in dynamic deviation with increase in contribution; higher interaction increasing deviation) between dynamic deviation and percentage anthropogenic contribution (R = 0.303, p < 0.05; not shown). The contributions from climatic variance might have reduced the impact of anthropogenic stress on water balance of the catchments.

The percentage contributions obtained from the eight Budyko based methods are distinguishable (Supplementary Table S6). The standard deviation is shown in Supplementary Fig. S4. Further, clustering of contributions from both parametric and nonparametric equations is observed because of the consideration of basin features that define the vegetation cover, topographical features and soil properties in parametric equations^9,29^. The difference between the mean of the contributions from the parametric and non-parametric equations (D) are obtained and the distribution is shown in Supplementary Fig. S5. The highest difference is seen in Shimoga (ID 28) with a value of 311.14%. From Supplementary Fig. S4, highest inconsistency in contributions is found in 16 catchments (IDs: 4, 7, 14, 15, 17, 18, 19, 24, 28, 34, 35, 37, 38, 51, 52 and 55). This inconsistency is explained by the trend line, changing its route and the slope was actually reduced indicating that ‘D’ slightly decreased in higher aridity index when plotted between summation of the absolute differences and summed aridity index during the baseline period (Fig. 4b**)**. Absolute differences were used to highlight more on the amount of change rather than the direction of change when parametric equations were used. The point shown with an arrow corresponds to aridity index value of 2.03. This could be the reason for high inconsistency for 15 catchments having aridity index value below 2.03 out of 16 as mentioned above, indicating the fact that basin characteristics played a major role in water balance of these catchments.

Sources of uncertainties in analysis may affect the accuracy of quantification of the parameters involved in the study. While developing the framework for contribution analysis, climatic variability and anthropogenic activities were considered to be independent of each other. However, the relationship between the two is very intricate and is never independent as they work together in hydrological systems. The long-term soil water storage changes are considered to be negligible during the implication of elasticity based method on Budyko hypothesis. But the parameter depends on the physiographical and soil properties of the catchments and there is no such firm evidence to approve the assumption^9^. Also while calculating annual mean AET for a 3-year time period using water balance equation, steady-state soil water storage is considered. But groundwater recharge and storages due to geological features and human interactions (inflows, withdrawals and water management techniques) alter the soil storages. The consideration of anthropogenic activities includes a vast domain of practices and therefore would always be better to differentiate the relative effects of it (e.g. foliage restoration, soil and water conservation measures, groundwater exploitation) on hydrological responses^37^. But this requires a huge amount of data which is one of the critical pitfalls for research in India. Further, analysis was carried out using the first order approximation of relative change in discharge due to climatic variance (Equation (6)). Therefore, errors may arise due to ignorance of higher orders of Taylor expansion in the study^38^. In addition, basin parameters of 4 parametric equations were considered constant throughout the time period (1988–2011) during contribution and resilience study. Results from pettitt test revealed non-stationarity of parameter ω, thus we acknowledge that considering the parameter as a variable (runoff elasticity to basin characteristics) may further enhance the contributions on runoff variability. However, differentiating the two periods were done on non-stationarity nature of ω, therefore considering a minor part of its impact in the study. Furthermore, human errors in gathering hydro-climatic data are responsible for additional uncertainties in the study.

The evolution of human civilisation has always been dependent on water yields from historical times. Therefore, predicting the alterations in water yield is one of the key to deal with the complications of variability of climatic conditions^39^. Moreover, the hydrology becomes more complicated or uncertain with the anthropogenic interactions^40,41^. A hydrological resilient catchment responds with water yields, within a predictable range under changing environments^12^. Our results indicated that human interactions had more influence than collective impact of P and ET in ecosystem on lowering the ability of the catchment to maintain partitioning of precipitation consistent with the Budyko curve. India is a vast country with an area of 3,287,590 km^2,17^ encompassing 2% of total world land area and constitutes approximately 17% of world population^42^. As per our findings, with future increase in population^43^, urbanisation and conversion of economic system and societal life^44^, it would impose more pressure on potential hydrologic resilience. This unpredictability nature of hydrological response can create disturbances in planning and establishment of infrastructures for water resources management projects^45^. Therefore, it calls for sustainable development and proper watershed management practises, to ameliorate the effects of anthropogenic stressors and continue relishing the benefits of ecosystem services.

## Methods

### Discharge data

Daily observed runoff data of 55 catchments during 1988–2011 are taken from India-WRIS WebGIS (Water Resources Information System) portal (http://www.india-wris.nrsc.gov.in/), which provides discharge dataset from Central Water Commission (CWC), Ministry of Water Resources, Govt. of India, New Delhi (India-WRIS, 2014).

### Meteorological data

The meteorological parameters including daily precipitation and minimum and maximum temperatures are obtained from Indian Meteorological Department (IMD)^46,47^.The precipitation data was available at high spatial resolution (0.25° × 0.25°). The data was available for study period from 1988 to 2011. The temperature data was available for the same period at spatial resolution of 1° × 1°. The average annual precipitation rasters are prepared using Inverse distance weighting interpolation method in ArcMap.

### Evaporative Index (EI) and Dryness Index (DI)

PET data was obtained from Climatic Research Unit (CRU) Time-series (TS) data version 4.01 data. CRU TS v. 4.01 provides gridded month-by-month variations in climate over the period 1901–2016 at high-resolution (0.5 × 0.5 degree) grids^48^. The dataset was produced using angular-distance weighting (ADW) interpolation. PET is calculated using a variant of the Penman–Monteith formula (http://www.fao.org/docrep/X0490E/x0490e06.htm), which takes into account different climatic variables such as mean temperature, maximum and minimum temperatures, vapour pressure, cloud cover and wind speed^48^. For the calculation of annual mean EI over a 3-year time period in resilience study, water balance equation (E = P – Q) is used with annual average P and Q as per an assumption of steady-state water soil storage (i.e. ΔS = 0) to quantify annual mean AET.

### Budyko Framework

The Budyko hypothesis conceptualises the long-term water balance at the catchment scale. The water balance equation over a long-term period is given as:

1 $$P=E+Q+{\rm{\Delta }}S$$

Here, P is mean annual precipitation, Q is mean annual runoff depth from the catchment, E is mean annual actual evapotranspiration and ΔS is the change in water storage. Generally, over a long time period (5–10 years), ΔS can be negligible (ΔS = 0). Budyko (1974)^49^ established a relationship between mean annual evaporation ratio (E/P) and aridity index (φ) i.e. E_o_/P (E_o_ is mean annual PET) given below:

2 $$\frac{E}{P}={[(1-{e}^{\frac{-{E}_{0}}{P}})\frac{{E}_{0}}{P}tanh{(\frac{{E}_{0}}{P})}^{-1}]}^{0.5}$$

The model works on the theory of water and energy limits; in humid state i.e. φ < 1, the evapotranspiration is limited by total available energy and in arid state i.e. φ > 1, water availability limits the evapotranspiration process. Over the years, researchers have introduced the impacts of basin characteristics like vegetation type, soil properties and topographical features into water balance and postulated mathematical functional forms with a parameter to explain the Budyko framework^29–32,36,50,51^. These parameters can be calculated from long term discharge, precipitation and PET values by considering negligible water storage change, using the corresponding Budyko type equations (Table 1**)**.

### Climate Elasticity Analysis

To date, two methods have been developed to quantify the contribution of anthropogenic activities and climate variability on runoff change using Budyko type equations^52^: Elasticity or sensitivity based method^53,54^ and Decomposition method^55^. The elasticity based method uses elasticity coefficients of runoff to assess the sensitivity of runoff change to variation in meteorological parameters whereas the decomposition method estimates the relative contributions independently without any sensitivity coefficients. Schaake^53^ proposed the elasticity based method to evaluate the impacts of climatic variability on runoff change. Elasticity-based method includes non-parametric and analytical method^54^. In the non-parametric method, the elasticity coefficients are established empirically from observed hydro-meteorological data. Arora^56^ suggested an analytical elasticity method based on Budyko hypothesis for assessing the impacts of precipitation and evaporation to runoff changes.

The long-term water balance, neglecting the change in water storage is given below^57^:

3 $$Q=P-E=P-Pf(\rlap{/}{0})$$

Considering the parameters P, E_o_ to be independent variables, the total differential indicating the change in runoff due to climatic variance is expressed as:

4 $$d{Q}_{C}=\frac{\partial {Q}_{C}}{\partial P}dP+\frac{\partial {Q}_{C}}{\partial {E}_{0}}d{E}_{0}$$

The relative change in runoff is then given as:

5 $$\frac{d{Q}_{C}}{Q}=(\frac{P}{Q}\frac{\partial {Q}_{C}}{\partial P})\frac{dP}{P}+(\frac{{E}_{0}}{Q}\frac{\partial {Q}_{C}}{\partial {E}_{0}})\frac{d{E}_{0}}{{E}_{0}}$$

This can be rewritten as:

6 $$\frac{d{Q}_{C}}{Q}={{\epsilon }}_{P}\frac{dP}{P}+{{\epsilon }}_{{E}_{0}}\frac{d{E}_{0}}{{E}_{0}}$$

where ${{\epsilon }}_{P}$ and ${{\epsilon }}_{{E}_{0}}$ are the elasticity coefficients of runoff to precipitation and potential evapotranspiration respectively.

From Equation (5) and using the water balance, the elasticity coefficients can be computed to be as follows:

7 $${{\epsilon }}_{P}=1+\frac{\rlap{/}{0}{f}^{\text{'}}(\rlap{/}{0})}{1-f(\rlap{/}{0})},\,{{\epsilon }}_{{E}_{0}}=\frac{-\rlap{/}{0}{f}^{\text{'}}(\rlap{/}{0})}{1-f(\rlap{/}{0})}$$

It can be seen that ${{\epsilon }}_{P}+{{\epsilon }}_{{E}_{0}}=1$. Therefore, runoff changes due to climatic variability can be expressed as:

8 $${\rm{\Delta }}{Q}_{C}=({{\epsilon }}_{P}\frac{{\rm{\Delta }}P}{P}+{{\epsilon }}_{{E}_{0}}\frac{{\rm{\Delta }}{E}_{0}}{{E}_{0}})Q$$

where, ΔP and ΔE_0_ are the changes in mean annual precipitation and potential evapotranspiration from baseline to assessment period.

Here, ${\in }_{P}\frac{{\rm{\Delta }}P}{P}Q$, and ${\in }_{{E}_{0}}\frac{{\rm{\Delta }}{E}_{0}}{{E}_{0}}Q$ are the changes in runoff due to precipitation and potential evapotranspiration variability respectively.

### Attribution Analysis of Changes in Runoff

The study period is divided into two time periods, the baseline period (1988–1997) and the assessment period (1998–2011). In this study, baseline period is taken as the first 10-year time period. Generally, baseline period defines a period with less anthropogenic activities. Since the degree of urbanisation increased rapidly during 2001–2011 with emergence of new towns^22^, we considered 1998–2011 as the assessment period. The change in observed runoff is computed using equation 9.

9 $${\rm{\Delta }}{Q}_{T}={Q}_{A}-{Q}_{B}$$

Q_A_ and Q_B_ are the mean annual observed discharges for assessment and baseline period respectively. ΔQ_T_ is the change in runoff from baseline to assessment period. To calculate the portion of runoff change caused due to anthropogenic, ΔQ_C_ is subtracted from ΔQ_T_ as suggested below:

10 $${\rm{\Delta }}{Q}_{T}={\rm{\Delta }}{Q}_{C}+{\rm{\Delta }}{Q}_{H}$$

ΔQ_H_ is the change in runoff due to human activities. To compute the relative contributions, following expressions are used:

11 $${P}_{C}=\frac{{\rm{\Delta }}{Q}_{C}}{{\rm{\Delta }}{Q}_{T}}\times 100,\,{P}_{H}=\frac{{\rm{\Delta }}{Q}_{H}}{{\rm{\Delta }}{Q}_{T}}\times 100$$

P_C_ and P_H_ are the percentage change in runoff due to climate variability and anthropogenic activities.

### Theoretical Budyko Curve

Theoretical budyko curve illustrates the predictive perception of a catchment’s response to changing climate. Parametric Budyko equations bring basin characteristics into the Budyko framework that provides better demonstration of partitioning of precipitation. Therefore, out of 8 Budyko equations, the equation given by Fu *et al*.^36^ is selected to develop the theoretical curve. The parameter ‘ω’, calculated in contribution analysis is used, assuming it to be constant throughout the climatic periods.

### Contrary climatic periods: cool and warm period

Two 3-year time periods are considered for the resilience study of catchments such that the cool period falls in the baseline period and warm period falls in the assessment period of contribution analysis (Table 2). The cool period is defined as the 3-year minimum temperature in baseline period and is cooler than at least 2, 3-year time periods temperature by more than 1 standard deviation. The warmer period is defined as the period with maximum 3-year temperature in assessment period such that it is warmer than at least 6, 3-year time periods by more than 1 standard deviation. Thus, the periods represent climatic warming shift with maximum temperature difference.

### Resilience Indices: Dynamic Deviation (d) and Modified Elasticity (e_m_)

**Figure 6:**
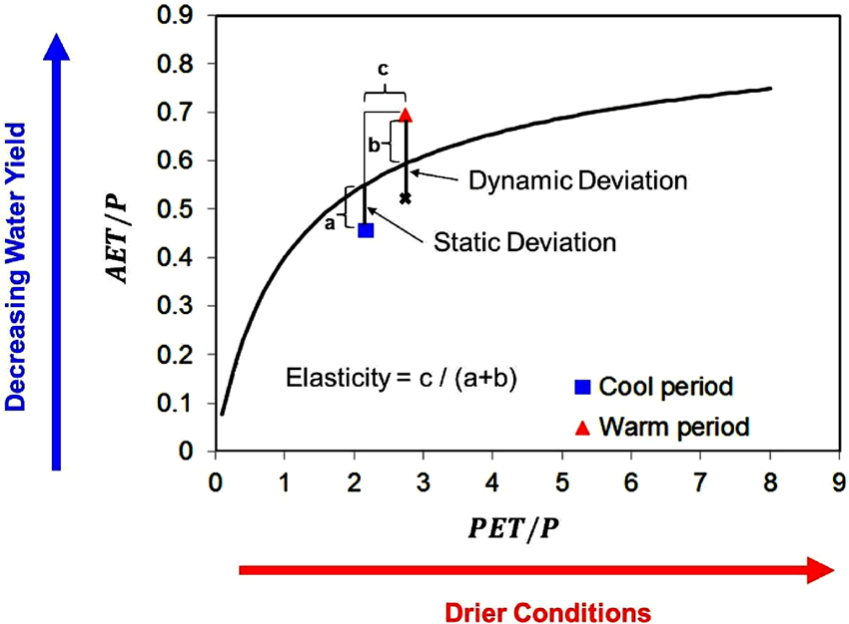
Representation of Budyko resilience metrics (dynamic deviation and modified elasticity). ‘a’ is EI_R,A_ (annual average Residual EI; departure of annual average EI from budyko curve) in cool period, ‘b’ is EI_R,A_ in warm period and c is range in DI_A_ (difference between average DIs from two periods). In the figure, ‘a’ denotes static deviation and the distance from cross mark to the red traingle mark denotes dynamic deviation (d = EI_M, W_ − EI_B, W_ − s). Modified elasticity (e_m_ = (DI_A, MAX_ − DI_A, MIN_)/(EI_R, A, MAX_ − EI_R, A,MIN_)) can be calculated by taking ratio of ‘c’ and sum of ‘a’ and ‘b’, as per the figure. Note that EI_R, A, MAX_ and EI_R, A,MIN_ denotes the maximum and minimum departure of annual average EIs from the theoritical budyko curve respectively.

Creed *et al*.^12^ introduced two indices i.e. dynamic deviation and elasticity. Deviation was summarized as the departure of the measured catchment’s EI (EI_M_) from the theoretical Budyko curve (EI_B_). It has two components: static deviation (s) and dynamic deviation (d). Static deviation denotes the departure of mean annual EI during the cool period (denoted by suffix C) due to the intrinsic catchment characteristics (s = EI_M, C_ − EI_B, C_). It was assumed to be unchanged throughout the period of study. Dynamic deviation (d) denotes the extent of deviation of mean annual EI during the climatic warm period (denoted by suffix W) from the theoretical budyko curve after correcting for static deviation (d = EI_M, W_ − EI_B, W_ − s) (Fig. 6). Thus, it represents virtuously the response to climate warming (not including the inherent characteristics). A positive (negative) d value would indicate reduction (increment) in water yield from the expected value (Fig. 6).

Elasticity (e) was defined as the ratio of inter-annual range in DI to inter-annual range in residual EI (e = (DI_MAX_ − DI_MIN_)/(EI_R, MAX_ − EI_R, MIN_)). EI residuals are calculated as EI_R_ = EI_M_ − EI_B_. We modified the parameter by considering the points representing average annual values of DIs and EI_R_s from both the extreme periods (3 years cool and warm period) over extreme inter-annual variability points. Average value signifies the influence of the climatic conditions as a whole. Our objective is not to consider the responses from exceptional weather cases that happen in a particular single year. We are more interested to see the response of the catchment to climate shifts and not to extreme events. Thus, our parameter, modified elasticity is defined as the ratio of the difference in annual average DIs to the difference between maximum and minimum deviation of annual average EI from the budyko curve (EI_R, A_; annual average residual EIs) from the two periods (e_m_ = (DI_A, MAX_ − DI_A, MIN_)/(EI_R, A, MAX_ − EI_R, A,MIN_)) as shown in Fig. 6. The subscript A denotes average values. Residual EI values are calculated to cope with the changing DI/EI ratio while going along the curve. A catchment with high value of modified elasticity, (smaller variation in EI_R_ relative to the variation in DI) partitions the precipitation to ET and runoff according to the theoretical Budyko curve (Fig. 6). The value of e_m_ = 1, has been taken as the threshold value for demarcation of elastic from non-elastic catchments.

## Electronic supplementary material

Supplementary Information

## Data Availability

The datasets generated during and/or analysed during the current study are available from the corresponding author on reasonable request.
