# Supplementary material for: Assessment of the impacts of climatic variability and anthropogenic stress on hydrologic resilience to warming shifts in Peninsular India
Source: Sci Rep. 2018 Sep 14;8:13833. doi: 10.1038/s41598-018-32091-0 (PMC6138737; doi:10.1038/s41598-018-32091-0)
Supplement: Supplementary file 1 — Supplementary Information [file 41598_2018_32091_MOESM1_ESM.docx]

**Assessment of the impacts of climatic variability and anthropogenic stress on hydrologic resilience to warming shifts in Peninsular India**

Jhilam Sinha^1^, Ashutosh Sharma^2^, Manas Khan^3^ and Manish Kumar Goyal^4*^

^1&4^Discipline of Civil Engineering, Indian Institute of Technology, Indore- 453552, India

^1,2,3&4^Dept. of Civil Engineering, Indian Institute of Technology, Guwahati -781039, India

(Corresponding author* email:vipmkgoyal@gmail.com)

# Supplementary Information

**Table S1** Annual mean values of hydro-meteorological parameters (precipitation (P), potential evapotranspiration (E_0_), runoff height (Q), aridity index (φ)) and area (km^2^) for all the catchments considered in the study.

**Table S2** Mann Kendall trend analysis of hydro-meteorological parameters. (This is followed by a text, discussing the trends)

**Table S3** Change points (CP) analysis of ω, P, Q and PET.

**Table S4** Aridity indices of the catchments during the study period.

**Table S5** Runoff elasticity coefficients to precipitation (ϵ_P_) and potential evapotranspiration (ϵ_E0_).

**Table S6** Relative contributions (%) of climate variability to runoff changes computed using eight Budyko-based methods.

**Table S7** Evapotranspiration elasticities to climatic parameters (Maximum temperature, minimum temperature, wind speed, relative humidity and sunshine duration).

**Fig. S1** Catchments considered on Peninsular India.

**Fig. S2** Spatial distribution of aridity indices and runoff coefficients.

**Fig. S3** Representation of catchmnent responses during cool and warm period with theoritical Budyko curve.

**Fig. S4** Dispersion of percentage contributions from climatic variability.

**Fig. S5** Spatial distribution of ‘D’ parameter.

|  |  |  |  | Mean annual values (1988-2011) | | | |
| --- | --- | --- | --- | --- | --- | --- | --- |
| ID | River Basin | Gauging Station | Area (km^2^) | P (mm) | E_0_ (mm) | Q (mm) | φ |
| 1 | Baitarani & | Anandapur | 8890.133 | 1544.159 | 1416.259 | 572.026 | 0.917 |
| 2 | Brahmani | Gomlai | 22340.55 | 1346.147 | 1440.779 | 503.027 | 1.070 |
| 3 |  | Jaraikela | 10708.86 | 1309.657 | 1372.977 | 429.453 | 1.048 |
| 4 |  | Tilga | 2684.779 | 1298.571 | 1376.85 | 742.84 | 1.060 |
| 5 | Cauvery | K.M.Vadi | 1454.402 | 1105.843 | 1423.984 | 234.817 | 1.288 |
| 6 |  | T.K.Halli | 8101.977 | 806.569 | 1547.897 | 93.58 | 1.919 |
| 7 |  | Thengumarahada | 673.26 | 1201.286 | 1448.84 | 491.34 | 1.206 |
| 8 | East Coast | Kashinagar | 8074.27 | 1327.354 | 1458.615 | 357.115 | 1.099 |
| 9 | Subzone | Purushottampur | 7154.389 | 1319.541 | 1458.254 | 304.568 | 1.105 |
| 10 |  | Srikakulam | 8633.538 | 1279.513 | 1472.547 | 312.098 | 1.151 |
| 11 | East Flowing | Thammavaram | 7992.959 | 852.309 | 1776.775 | 132.284 | 2.085 |
| 12 | Rivers | Vazhavachanur | 11594.07 | 820.246 | 1630.639 | 31.91 | 1.988 |
| 13 |  | A.P.Puram | 1286.371 | 880.479 | 1373.341 | 23.255 | 1.560 |
| 14 |  | Murappanadu | 2912.394 | 815.329 | 1391.695 | 191.088 | 1.707 |
| 15 |  | Theni | 899.245 | 1056.67 | 1331.776 | 684.78 | 1.260 |
| 16 | Godavari | Bhatpalli | 3134.297 | 1177.793 | 1736.081 | 406.051 | 1.474 |
| 17 |  | Hivra | 10187.12 | 910.345 | 1713.065 | 157.631 | 1.882 |
| 18 |  | Nandgaon | 4527.217 | 1058.779 | 1730.444 | 197.995 | 1.634 |
| 19 |  | Pachegaon | 5554.247 | 764.048 | 1623.578 | 134.638 | 2.125 |
| 20 |  | Purna | 15119.35 | 813.887 | 1738.094 | 116.35 | 2.136 |
| 21 |  | Satrapur | 7398.06 | 1044.75 | 1649.465 | 279.951 | 1.579 |
| 22 |  | Zari | 5524.573 | 806.434 | 1748.905 | 106.803 | 2.169 |
| 23 |  | P.G.Bridge | 14093.19 | 992.483 | 1744.017 | 327.463 | 1.682 |
| 24 |  | Pathagudem | 39148.24 | 1455.331 | 1560.577 | 596.853 | 1.058 |
| 25 | Krishna | Cholachguda | 9897.489 | 794.747 | 1640.778 | 95.743 | 2.065 |
| 26 |  | Phulgaon | 2117.593 | 1648.781 | 1546.705 | 640.2 | 0.938 |
| 27 |  | Sarati | 6754.838 | 963.935 | 1603.879 | 199.91 | 1.664 |
| 28 |  | Shimoga | 2727.495 | 2361.049 | 1491.12 | 2009.647 | 0.632 |
| 29 | Mahanadi | Andhiyarkore | 2141.483 | 1090.884 | 1545.391 | 161.159 | 1.417 |
| 30 |  | Ghatora | 2980.721 | 1264.888 | 1518.6 | 314.462 | 1.201 |
| 31 |  | Kotni | 7012.965 | 1207.071 | 1624.024 | 281.769 | 1.345 |
| 32 |  | Pathardhi | 2508.821 | 1152.145 | 1619.347 | 435.44 | 1.406 |
| 33 |  | Simga | 16906.83 | 1151.36 | 1620.103 | 285.441 | 1.407 |
| 34 | Mahi | Mataji | 3968.568 | 943.518 | 1826.646 | 356.688 | 1.936 |
| 35 | Narmada | Gadarwara | 2212.957 | 1120.166 | 1559.582 | 644.526 | 1.392 |
| 36 |  | Kogaon | 3887.818 | 802.987 | 1807.429 | 322.548 | 2.251 |
| 37 |  | Mohgaon | 4017.72 | 1209.037 | 1509.409 | 602.792 | 1.248 |
| 38 |  | Patan | 4038.501 | 1198.068 | 1494.529 | 423.888 | 1.247 |
| 39 | Pennar Basin | Alladupalli | 8697.704 | 793.134 | 1827.927 | 189.655 | 2.305 |
| 40 |  | Singavaram | 5921.54 | 651.191 | 1737.636 | 34.668 | 2.668 |
| 41 | Sabarmati | Kheroj | 878.144 | 722.25 | 1792.308 | 453.366 | 2.482 |
| 42 | Subanarekha | Adityapur | 6416.672 | 1388.369 | 1376.885 | 459.118 | 0.992 |
| 43 |  | Govindpur | 4324.758 | 1670.52 | 1385.213 | 732.235 | 0.829 |
| 44 | Tapi Basin | Gidhade | 52134.18 | 825.647 | 1728.894 | 142.15 | 2.094 |
| 45 | West Flowing | Gandhav | 60866.51 | 399.566 | 1929.564 | 3.688 | 4.829 |
| 46 | Rivers | Kamalpur | 5011.097 | 733.729 | 1855.281 | 78.374 | 2.529 |
| 47 |  | Ayilam | 502.036 | 1585.415 | 1378.625 | 1403.053 | 0.870 |
| 48 |  | Durvesh | 1990.403 | 2551.768 | 1611.251 | 1587.014 | 0.631 |
| 49 |  | Erinjipuzha | 852.844 | 3585.738 | 1484.831 | 2703.864 | 0.414 |
| 50 |  | Kallooppara | 697.304 | 2965.507 | 1393.928 | 2571.892 | 0.470 |
| 51 |  | Karathodu | 756.335 | 2277.392 | 1474.243 | 1747.503 | 0.647 |
| 52 |  | Kidangoor | 592.58 | 3024.344 | 1392.91 | 2915.956 | 0.461 |
| 53 |  | Mahuwa | 1712.581 | 1340.357 | 1695.616 | 829.624 | 1.265 |
| 54 |  | Thumpamon | 814.037 | 1854.197 | 1377.502 | 1472.098 | 0.743 |
| 55 |  | Gadat | 1465.974 | 1679.121 | 1676.603 | 1124.297 | 0.929 |

**Supplementary Table S1.** Annual mean values of hydro-meteorological parameters (precipitation (P), potential evapotranspiration (E_0_), runoff height (Q), aridity index (φ)) and area (km^2^) for all the catchments considered in the study

| ID | Q | | P | | E_0_ | |
| --- | --- | --- | --- | --- | --- | --- |
|  | Z | β(mm/yr) | Z | β(mm/yr) | Z | β(mm/yr) |
| 1 | -0.571 | -5.394 | 0.422 | 3.809 | -0.174 | -0.356 |
| 2 | -0.273 | -2.887 | -1.067 | -8.398 | 0.769 | 0.666 |
| 3 | -1.067 | -6.948 | 0.868 | 5.951 | 0.571 | 0.627 |
| 4 | -0.422 | -3.293 | -0.571 | -2.24 | 1.563 | 0.770 |
| 5 | 0.422 | 2.066 | 1.364 | 9.563 | -2.307* | -0.902 |
| 6 | 1.067 | 0.968 | 0.967 | 4.138 | -1.811 | -0.818 |
| 7 | 0.62 | 3.817 | -0.521 | -2.915 | -2.208* | -0.849 |
| 8 | 0.025 | 0.622 | -1.315 | -7.77 | 0.521 | 0.702 |
| 9 | 0.025 | 0.064 | -0.273 | -2.37 | 0.372 | 0.537 |
| 10 | -0.918 | -4.151 | 0.025 | 0.721 | 0.719 | 0.583 |
| 11 | -1.612 | -3.206 | 0.67 | 4.137 | -0.174 | -0.167 |
| 12 | 0.273 | 0.223 | 0.719 | 3.438 | -1.712 | -0.785 |
| 13 | 0.62 | 0.124 | 1.166 | 7.477 | -2.356* | -0.864 |
| 14 | 0.521 | 1.122 | 1.265 | 6.843 | -2.108* | -0.845 |
| 15 | -0.074 | -0.478 | 2.059* | 12.835 | -1.463 | -0.752 |
| 16 | -2.555* | -16.845 | -0.521 | -6.541 | 1.315 | 0.717 |
| 17 | -0.223 | -0.322 | -0.273 | -1.07 | 1.712 | 0.926 |
| 18 | 0.074 | 0.854 | -0.074 | -0.514 | 1.811 | 1.087 |
| 19 | -0.074 | -0.655 | 1.116 | 6.667 | 0.967 | 0.335 |
| 20 | -1.612 | -3.141 | -0.967 | -4.3 | 0.868 | 0.379 |
| 21 | -1.315 | -4.812 | 0.074 | 0.649 | 1.811 | 0.984 |
| 22 | -2.456* | -4.107 | -1.017 | -6.463 | 0.521 | 0.252 |
| 23 | -1.463 | -10.652 | -0.967 | -10.504 | 1.265 | 0.529 |
| 24 | -0.571 | -3.007 | -0.67 | -4.833 | 0.967 | 0.692 |
| 25 | -0.819 | -1.174 | 1.315 | 7.322 | -0.620 | -0.271 |
| 26 | -1.662 | -13.584 | 1.265 | 25.67 | 0.422 | 0.153 |
| 27 | -1.017 | -4.702 | 1.215 | 9.01 | -0.124 | -0.047 |
| 28 | 0.521 | 6.071 | 1.712 | 29.091 | -1.712 | -0.549 |
| 29 | -1.96 | -4.549 | 0.322 | 2.185 | 1.662 | 1.026 |
| 30 | -2.679** | -12.196 | -0.273 | -3.346 | 1.463 | 1.024 |
| 31 | 0.769 | 3.85 | 0.074 | 1.409 | 1.414 | 0.956 |
| 32 | -1.166 | -6.908 | 0.223 | 1.223 | 1.414 | 1.055 |
| 33 | 0.273 | 1.573 | 0.223 | 2.654 | 1.513 | 1.005 |
| 34 | -0.074 | -1.015 | -0.719 | -5.664 | 1.563 | 1.014 |
| 35 | 0.273 | 3.025 | -0.223 | -3.644 | 1.860 | 1.168 |
| 36 | -1.414 | -9.393 | -0.868 | -4.67 | 1.364 | 0.596 |
| 37 | -0.67 | -5.039 | -1.067 | -10.011 | 1.761 | 1.129 |
| 38 | -0.273 | -0.976 | -0.471 | -5.411 | 2.009* | 0.967 |
| 39 | 1.315 | 4.411 | 0.471 | 2.128 | -0.769 | -0.414 |
| 40 | 2.108* | 0.841 | 0 | 0.171 | -1.364 | -0.731 |
| 41 | -0.62 | -8.744 | -0.372 | -1.862 | 1.315 | 1.221 |
| 42 | -0.62 | -5.557 | 0.769 | 7.72 | 0.025 | 0.006 |
| 43 | 1.513 | 12.026 | -0.422 | -3.581 | -0.571 | -0.521 |
| 44 | -1.166 | -2.221 | -0.422 | -3.17 | 1.265 | 0.638 |
| 45 | -2.059** | -0.192 | -0.074 | -0.799 | 0.571 | 0.573 |
| 46 | 0.124 | 0.008 | -0.273 | -1.403 | 1.662 | 1.451 |
| 47 | -0.62 | -11.617 | 0.322 | 4.757 | -2.257* | -0.982 |
| 48 | 2.853** | 47.609 | 1.463 | 18.604 | 0.471 | 0.207 |
| 49 | -0.62 | -10.684 | -1.166 | -22.026 | -2.257* | -0.802 |
| 50 | -0.769 | -12.634 | 0.868 | 23.313 | -1.960 | -0.798 |
| 51 | 0.124 | 2.871 | 1.563 | 28.371 | -1.811 | -0.906 |
| 52 | -0.372 | -9.734 | 1.414 | 32.691 | -1.761 | -0.753 |
| 53 | 0.273 | 5.796 | -1.067 | -9.627 | 1.513 | 0.777 |
| 54 | -1.414 | -19.398 | 1.265 | 17.084 | -2.208* | -0.768 |
| 55 | 1.166 | 18.623 | -1.265 | -14.879 | 1.315 | 0.737 |

**Supplementary Table S2.** Mann Kendall trend analysis of hydro-meteorological parameters. The table highlights the existing trends in P, ET_0_ and Q with Z statistics and significance of Non-parametric Mann Kendall test and magnitude of the trend (slope) by Sen’s slope estimator test for 55 catchments during the period of 24 years (1988-2011). * and ** indicate the significance level of 0.05 and 0.01 respectively.

Text for discussion of the trend analysis result (**Supplementary Table S2**):

- The annual runoff generation showed a downward trend in 34 catchments though only 4 of them are significant (IDs: 16 (p < 0.05), 22 (p < 0.05), 30 (p < 0.01), 45 (p<0.01)). Rest of the catchments showed increasing rate with 2 having a significant upward trend (IDs: 40 (P < 0.05) and 48 (p<0.01)). The decreasing rate ranges from -13.58 mm/yr (ID: 26) to -0.02 mm/yr (ID: 45).
- The annual precipitation showed a very varied changing rate with 26 catchments having downward trends and 29 catchments showing increasing trends. None of them were significant except catchment Theni (p < 0.05) that showed a significant increasing trend.
- Like precipitation, annual E_0_ rate varied with 21 catchments showing downward trend with 7 catchments having significant downward trend (IDs: 5 (p < 0.05), 7 (p < 0.05), 13 (p < 0.05), 14 (p< 0.05), 47 (p < 0.05), 49 (p< 0.05), 54 (p< 0.05)). Only one catchment had significant upward trend (ID 38 (p < 0.05)) among 34 upward trend catchments.

| **Catchment name** | **Change Point** | | | |
| --- | --- | --- | --- | --- |
|  | **w** | **P** | **Q** | **PET** |
| Anandapur | 2000** | NS | NS | NS |
| Gomlai | NS | NS | NS | NS |
| Jaraikela | 2002** | NS | NS | NS |
| Tilga | NS | NS | NS | 1995* |
| K.M.Vadi | 1997** | NS | NS | 2004** |
| T.K.Halli | NS | NS | NS | 2003** |
| Thengumarahada | 2001** | NS | NS | 2003** |
| Kashinagar | 2003** | NS | NS | NS |
| Purushottampur | 2002** | NS | NS | NS |
| Srikakulam | 1999** | NS | NS | NS |
| Thammavaram | 2001** | NS | 2000** | NS |
| Vazhavachanur | 2005* | NS | NS | 2003** |
| A.P.Puram | 2005** | 2003* | NS | 2003** |
| Murappanadu | NS | 2003* | NS | 2003** |
| Theni | 2001** | 2004** | NS | 2003** |
| Bhatpalli | 2002** | NS | 2000* | NS |
| Hivra | NS | NS | NS | NS |
| Nandgaon | NS | NS | NS | 1999* |
| Pachegaon | NS | NS | NS | NS |
| Purna | NS | NS | NS | NS |
| Satrapur | 2000** | NS | NS | 1999* |
| Zari | 2002** | NS | NS | NS |
| P.G.Bridge | NS | NS | NS | NS |
| Pathagudem | 2005** | NS | NS | NS |
| Cholachguda | 2001** | 2003* | NS | NS |
| Phulgaon | 2002** | 2003** | NS | NS |
| Sarati | 2000** | 2003* | NS | NS |
| Shimoga | 2002** | 2003** | NS | 2003** |
| Andhiyarkore | 1999** | NS | 2005* | 1995* |
| Ghatora | 2001** | NS | 1999* | 1995* |
| Kotni | 2005* | NS | NS | NS |
| Pathardhi | NS | NS | NS | 1995** |
| Simga | NS | NS | NS | 1995* |
| Mataji | 2004** | NS | NS | NS |
| Gadarwara | 2004** | NS | NS | 1999** |
| Kogaon | NS | NS | NS | NS |
| Mohgaon | 2002* | NS | NS | 1995* |
| Patan | NS | NS | NS | NS |
| Alladupalli | 1998** | NS | NS | 2004** |
| Singavaram | 2000** | NS | NS | 2003** |
| Kheroj | 1996** | NS | NS | NS |
| Adityapur | 2000** | NS | NS | NS |
| Govindpur | 1999** | NS | NS | NS |
| Gidhade | NS | NS | NS | NS |
| Gandhav | NS | NS | 1995* | NS |
| Kamalpur | 2002** | NS | NS | NS |
| Ayilam | NS | NS | NS | NS |
| Durvesh | 2000** | NS | 2001** | NS |
| Erinzipuzha | 2003* | NS | NS | 2004* |
| Kallooppara | 1998** | NS | NS | 2003* |
| Karathodu | 1996** | NS | NS | 2003** |
| Kidangoor | 2000** | NS | NS | 2003* |
| Mahuwa | 2002** | NS | NS | NS |
| Thumpamon | 1998** | NS | NS | 1998* |
| Gadat | 2003** | NS | NS | NS |

**Supplementary Table S3:** Change points (CP) analysis of ω, P, Q and PET. ** and * signifies CP at 95% and 90% significance level respectively. NS shows non-significant change points.

Text for discussion of the time series in change point analysis (**Supplementary Table S3**):

A time series of ω is generated using Budyko equation^1^ applying moving average method taking a time window of 5 years for each of the 55 river basins for the time span of 1988-2011. Selection of proper time window is critical but there is no specific rule^2^. Thus, time series of ω is generated for 20 years starting from 1990 to 2009. For P, PET and Q, time series is from 1988-2011. Pettitt test is also applied in previous studies for change detection^3,4^

1. Fu, G., Charles, S. P. & Chiew, F. H. S. A two-parameter climate elasticity of streamflow index to assess climate change effects on annual streamflow. *Water Resour. Res.* **43,** 1–12 (2007).

2. Jiang, C. *et al.* Separating the impacts of climate change and human activities on runoff using the Budyko-type equations with time-varying parameters. *J. Hydrol.* **522,** 326–338 (2015).

3. Yong, B. *et al.* Spatial–Temporal Changes of Water Resources in a Typical Semiarid Basin of North China over the Past 50 Years and Assessment of Possible Natural and Socioeconomic Causes. *J. Hydrometeorol.* **14,** 1009–1034 (2013).

4. Wang, W. *et al.* Quantitative assessment of the impact of climate variability and human activities on runoff changes: A case study in four catchments of the Haihe River basin, China. *Hydrol. Process.* **27,** 1158–1174 (2013).

| Catchment ID | φ (Baseline period) | φ (Assessment period) | Change in φ |
| --- | --- | --- | --- |
| 1 | 0.922 | 0.914 | -0.008 |
| 2 | 1.052 | 1.083 | 0.031 |
| 3 | 1.056 | 1.043 | -0.013 |
| 4 | 1.013 | 1.096 | 0.083 |
| 5 | 1.324 | 1.263 | -0.062 |
| 6 | 1.983 | 1.876 | -0.108 |
| 7 | 1.136 | 1.262 | 0.126 |
| 8 | 1.023 | 1.159 | 0.136 |
| 9 | 1.055 | 1.144 | 0.089 |
| 10 | 1.147 | 1.153 | 0.006 |
| 11 | 2.051 | 2.110 | 0.059 |
| 12 | 1.977 | 1.996 | 0.020 |
| 13 | 1.620 | 1.519 | -0.101 |
| 14 | 1.769 | 1.665 | -0.104 |
| 15 | 1.374 | 1.189 | -0.185 |
| 16 | 1.425 | 1.511 | 0.086 |
| 17 | 1.818 | 1.930 | 0.112 |
| 18 | 1.585 | 1.671 | 0.086 |
| 19 | 2.584 | 1.886 | -0.698 |
| 20 | 2.080 | 2.177 | 0.098 |
| 21 | 1.564 | 1.589 | 0.025 |
| 22 | 2.099 | 2.221 | 0.123 |
| 23 | 1.583 | 1.759 | 0.176 |
| 24 | 0.988 | 1.114 | 0.126 |
| 25 | 2.318 | 1.915 | -0.404 |
| 26 | 1.199 | 0.812 | -0.387 |
| 27 | 2.037 | 1.471 | -0.566 |
| 28 | 0.670 | 0.607 | -0.063 |
| 29 | 1.466 | 1.384 | -0.082 |
| 30 | 1.143 | 1.245 | 0.103 |
| 31 | 1.295 | 1.383 | 0.088 |
| 32 | 1.352 | 1.446 | 0.093 |
| 33 | 1.379 | 1.427 | 0.048 |
| 34 | 1.765 | 2.078 | 0.313 |
| 35 | 1.356 | 1.419 | 0.063 |
| 36 | 2.162 | 2.318 | 0.156 |
| 37 | 1.203 | 1.283 | 0.080 |
| 38 | 1.184 | 1.296 | 0.112 |
| 39 | 2.336 | 2.283 | -0.054 |
| 40 | 2.585 | 2.732 | 0.147 |
| 41 | 2.249 | 2.677 | 0.429 |
| 42 | 1.003 | 0.984 | -0.020 |
| 43 | 0.800 | 0.851 | 0.051 |
| 44 | 2.026 | 2.146 | 0.120 |
| 45 | 4.302 | 5.287 | 0.984 |
| 46 | 2.270 | 2.750 | 0.480 |
| 47 | 0.852 | 0.883 | 0.031 |
| 48 | 0.668 | 0.608 | -0.060 |
| 49 | 0.391 | 0.433 | 0.042 |
| 50 | 0.497 | 0.452 | -0.044 |
| 51 | 0.683 | 0.624 | -0.059 |
| 52 | 0.488 | 0.443 | -0.045 |
| 53 | 1.218 | 1.301 | 0.083 |
| 54 | 0.778 | 0.719 | -0.059 |
| 55 | 0.883 | 0.965 | 0.082 |

**Supplementary Table S4:** Aridity indices of the catchments during the study period. The baseline period is considered the first 10 years of study period (1988 to 1997) whereas, assessment period is taken from 1998 to 2011.

| Catchment ID | ϵ_P_ | ϵ_E0_ |
| --- | --- | --- |
| 1 | 2.021 | -1.021 |
| 2 | 2.037 | -1.037 |
| 3 | 2.115 | -1.115 |
| 4 | 1.958 | -0.958 |
| 5 | 2.390 | -1.390 |
| 6 | 2.675 | -1.675 |
| 7 | 2.013 | -1.013 |
| 8 | 2.248 | -1.248 |
| 9 | 2.355 | -1.355 |
| 10 | 2.311 | -1.311 |
| 11 | 2.579 | -1.579 |
| 12 | 3.056 | -2.056 |
| 13 | 3.397 | -2.397 |
| 14 | 2.369 | -1.369 |
| 15 | 2.025 | -1.025 |
| 16 | 2.153 | -1.153 |
| 17 | 2.516 | -1.516 |
| 18 | 2.463 | -1.463 |
| 19 | 2.537 | -1.537 |
| 20 | 2.614 | -1.614 |
| 21 | 2.291 | -1.291 |
| 22 | 2.644 | -1.644 |
| 23 | 2.211 | -1.211 |
| 24 | 1.979 | -0.979 |
| 25 | 2.669 | -1.669 |
| 26 | 1.991 | -0.991 |
| 27 | 2.419 | -1.419 |
| 28 | 1.575 | -0.575 |
| 29 | 2.583 | -1.583 |
| 30 | 2.297 | -1.297 |
| 31 | 2.336 | -1.336 |
| 32 | 2.095 | -1.095 |
| 33 | 2.309 | -1.309 |
| 34 | 2.409 | -1.409 |
| 35 | 2.111 | -1.111 |
| 36 | 2.492 | -1.492 |
| 37 | 2.086 | -1.086 |
| 38 | 2.099 | -1.099 |
| 39 | 2.446 | -1.446 |
| 40 | 2.956 | -1.956 |
| 41 | 2.475 | -1.475 |
| 42 | 2.106 | -1.106 |
| 43 | 1.889 | -0.889 |
| 44 | 2.542 | -1.542 |
| 45 | 3.437 | -2.437 |
| 46 | 2.750 | -1.750 |
| 47 | 1.742 | -0.742 |
| 48 | 1.667 | -0.667 |
| 49 | 1.422 | -0.422 |
| 50 | 1.431 | -0.431 |
| 51 | 1.613 | -0.613 |
| 52 | 1.399 | -0.399 |
| 53 | 2.038 | -1.038 |
| 54 | 1.677 | -0.677 |
| 55 | 1.842 | -0.842 |

**Supplementary Table S5.** Runoff elasticity coefficients to precipitation (ϵ_P_) and potential evapotranspiration (ϵ_E0_).

| ID | BDK-S | BDK-O | BDK | BDK-PT | BDK-FY | BDK-Z | BDK-CY | BDK-WT |
| --- | --- | --- | --- | --- | --- | --- | --- | --- |
| 1* | -17.68 h | -20.83 h | -18.91 h | -19.21 h | -17.66 h | -17.69 h | -17.63 h | -17.70 h |
| 2* | 42.30 h | 52.10 | 46.08 h | 46.88 h | 35.83 h | 37.18 h | 35.90 h | 35.80 h |
| 3* | -13.85 h | -16.12 h | -14.73 h | -14.92 h | -13.49 h | -13.59 h | -13.51 h | -13.47 h |
| 4* | 251.14 | 304.79 | 271.85 | 276.33 | 161.87 | NA | 162.06 | NA |
| 5* | -64.98 h | -74.83 h | -68.80 h | -68.79 h | -67.67 h | -65.41 h | -68.35 h | -65.70 h |
| 6 | 99.13 | 95.11 | 98.15 | 90.51 | 86.97 | 86.02 | 89.60 | 79.12 |
| 7* | 410.69 | 476.50 | 436.08 | 438.88 | 308.29 | 317.38 | 309.76 | 309.16 |
| 8* | 181.19 | 217.44 | 195.17 | 197.92 | 192.72 | 187.20 | 193.32 | 191.14 |
| 9* | 235.36 | 282.76 | 253.63 | 257.17 | 278.36 | 256.23 | 279.34 | 274.39 |
| 10* | 2.02 h | 3.16 h | 2.46 h | 2.53 h | 2.52 h | 2.26 h | 2.55 h | 2.42 h |
| 11* | 20.94 h | 19.19 h | 20.40 h | 18.38 h | 14.94 h | 15.97 h | 15.41 h | 14.31 h |
| 12* | 12.55 h | 11.95 h | 12.38 h | 11.50 h | 14.94 h | 12.05 h | 15.36 h | 11.64 h |
| 13* | -44.99 h | -47.95 h | -46.26 h | -44.82 h | -92.96 h | -51.43 h | -95.09 h | -61.00 h |
| 14* | -769.11 h | -784.83 h | -778.42 h | -739.23 h | -556.16 h | -596.39 h | -568.24 h | -549.31 h |
| 15* | -269.20 h | -310.52 h | -285.21 h | -285.84 h | -145.94 h | NA | -146.53 h | NA |
| 16* | 26.51 h | 28.96 h | 27.50 h | 26.94 h | 17.95 h | 18.90 h | 18.18 h | 18.05 h |
| 17* | 388.44 | 376.54 | 386.02 | 357.51 | 292.97 | 310.19 | 301.12 | 281.80 |
| 18 | -733.36 h | -765.03 h | -748.21 h | -717.30 h | -621.40 h | -639.53 h | -634.87 h | -597.29 h |
| 19 | 697.91 | 633.79 | 677.47 | 608.33 | 468.19 | 507.84 | 482.56 | 456.87 |
| 20* | 153.68 | 139.01 | 148.98 | 133.40 | 110.71 | 117.71 | 114.33 | 105.18 |
| 21* | 10.44 h | 11.22 h | 10.78 h | 10.33 h | 6.93 h | 7.58 h | 7.10 h | 6.88 h |
| 22* | 73.99 | 66.37 | 71.51 | 63.77 | 54.07 | 57.10 | 55.88 | 50.92 |
| 23* | 109.22 | 112.17 | 110.78 | 105.77 | 68.65 | 72.13 | 69.83 | 69.40 |
| 24* | 390.24 | 472.35 | 421.94 | 428.81 | 318.45 | 329.10 | 318.92 | 318.53 |
| 25* | -386.67 h | -356.84 h | -377.53 h | -341.81 h | -309.10 h | -316.55 h | -319.01 h | -285.70 h |
| 26* | -209.36 h | -257.16 h | -227.99 h | -232.51 h | -197.52 h | -200.83 h | -197.21 h | -197.78 h |
| 27* | -321.47 h | -331.67 h | -326.57 h | -312.76 h | -257.68 h | -270.29 h | -262.85 h | -251.46 h |
| 28* | -695.70 h | -861.80 h | -764.39 h | -777.21 h | -464.42 h | NA | -462.85 h | NA |
| 29* | -53.76 h | -58.99 h | -55.84 h | -55.16 h | -60.49 h | -55.39 h | -61.34 h | -56.74 h |
| 30* | 36.03 h | 42.38 h | 38.48 h | 38.76 h | 36.72 h | 36.09 h | 36.96 h | 36.13 h |
| 31* | 90.58 | 102.54 | 95.26 | 94.70 | 84.67 | 85.44 | 85.64 | 82.84 |
| 32* | 44.57 h | 49.64 h | 46.58 h | 46.00 h | 29.88 h | 31.04 h | 30.21 h | 30.10 h |
| 33* | 48.56 h | 54.38 | 50.87 | 50.19 | 41.31 h | 42.88 h | 41.89 h | 40.59 h |
| 34* | 260.73 | 249.13 | 257.76 | 237.41 | 135.48 | NA | 138.26 | NA |
| 35 | -264.24 h | -296.81 h | -277.13 h | -273.95 h | -133.35 h | NA | -134.43 h | NA |
| 36* | 66.45 | 58.13 | 63.64 | 55.93 | 29.21 h | NA | 29.94 h | NA |
| 37* | 223.37 | 260.68 | 237.81 | 238.63 | 136.78 | NA | 137.58 | NA |
| 38* | 290.41 | 337.42 | 308.60 | 309.66 | 225.85 | 236.50 | 227.43 | 225.91 |
| 39 | 18.62 h | 16.12 h | 17.76 h | 15.54 h | 10.21 h | 11.10 h | 10.53 h | 10.34 h |
| 40 | -171.18 h | -135.68 h | -157.73 h | -131.99 h | -133.01 h | -129.16 h | -138.55 h | -111.02 h |
| 41* | 112.63 | 92.76 | 105.45 | 89.79 | 37.83 h | NA | 38.50 h | NA |
| 42* | -16.29 h | -19.58 h | -17.56 h | -17.87 h | -16.50 h | -16.43 h | -16.50 h | -16.51 h |
| 43 | -51.32 h | -63.88 h | -56.29 h | -57.46 h | 48.63 h | -49.36 h | -48.44 h | -48.74 h |
| 44* | 72.54 | 66.26 | 70.58 | 63.46 | 49.22 h | 53.31 | 50.77 | 47.78 h |
| 45* | 70.84 | 35.76 h | 48.44 h | 35.41 h | 38.90 h | 35.40 h | 40.70 h | 26.76 h |
| 46* | 59.75 | 48.60 h | 55.67 | 47.09 h | 39.49 h | 42.28 h | 41.12 h | 36.57 h |
| 47* | 73.26 | 88.66 | 79.32 | 80.77 | 45.51 h | NA | 45.48 h | NA |
| 48 | 42.93 h | 52.98 | 47.09 h | 47.86 h | 36.28 h | NA | 36.06 h | NA |
| 49* | 128.14 | 147.48 | 136.70 | 138.20 | 111.59 | NA | 110.87 | NA |
| 50* | -88.89 h | -106.06 h | -96.34 h | -97.57 h | -64.91 h | NA | -64.60 h | NA |
| 51* | -242.26 h | -301.76 h | -266.77 h | -271.44 h | -170.29 h | NA | -169.51 h | NA |
| 52* | -209.03 h | -248.25 h | -226.12 h | -228.94 h | -143.24 h | NA | -142.99 h | NA |
| 53 | -106.02 h | -122.26 h | -112.31 h | -112.52 h | -58.52 h | NA | -58.78 h | NA |
| 54* | -68.49 h | -86.42 h | -75.71 h | -77.27 h | -43.26 h | NA | -43.13 h | NA |
| 55 | -303.82 h | -375.14 h | -331.65 h | -338.41 h | -195.20 h | NA | -194.98 h | NA |

**Supplementary Table S6.** Relative contributions (%) of climate variability to runoff changes computed using eight Budyko-based methods. * indicates that the catchments have experienced decreasing runoff generation from baseline to assessment period. –ve sign denotes that the contribution is opposite to the direction, runoff has changed. The values with ‘h’ indicate larger impact of anthropogenic activities on runoff changes.

| Catchment ID | Evapotranspiration elasticities to climatic parameters | | | | |
| --- | --- | --- | --- | --- | --- |
|  | Max Temperature | Min Temperature | Wind speed | Relative humidity | Sunshine duration |
| 1 | -0.00024 | -0.00014 | -3.20158 | 0.00002 | 0.00015 |
| 2 | -0.00024 | -0.00014 | -3.12057 | 0.00002 | 0.00014 |
| 3 | -0.00025 | -0.00014 | -3.12137 | 0.00002 | 0.00015 |
| 4 | -0.00023 | -0.00012 | -2.88591 | 0.00002 | 0.00016 |
| 5 | -0.00025 | -0.00015 | -3.22044 | 0.00002 | 0.00025 |
| 6 | -0.00024 | -0.00014 | -3.14352 | 0.00002 | 0.00021 |
| 7 | -0.00025 | -0.00015 | -3.26856 | 0.00002 | 0.00026 |
| 8 | -0.00025 | -0.00016 | -3.24216 | 0.00002 | 0.00019 |
| 9 | -0.00026 | -0.00017 | -3.25370 | 0.00002 | 0.00018 |
| 10 | -0.00026 | -0.00016 | -3.31185 | 0.00002 | 0.00019 |
| 11 | -0.00027 | -0.00017 | -3.61496 | 0.00002 | 0.00015 |
| 12 | -0.00023 | -0.00014 | -3.18877 | 0.00001 | 0.00019 |
| 13 | -0.00025 | -0.00017 | -3.02928 | 0.00001 | 0.00023 |
| 14 | -0.00027 | -0.00019 | -3.38554 | 0.00001 | 0.00021 |
| 15 | -0.00024 | -0.00016 | -2.96169 | 0.00001 | 0.00026 |
| 16 | -0.00029 | -0.00017 | -3.62534 | 0.00003 | 0.00015 |
| 17 | -0.00029 | -0.00017 | -3.52259 | 0.00002 | 0.00013 |
| 18 | -0.00029 | -0.00017 | -3.57042 | 0.00002 | 0.00013 |
| 19 | -0.00029 | -0.00015 | -3.25452 | 0.00002 | 0.00019 |
| 20 | -0.00028 | -0.00015 | -3.35584 | 0.00002 | 0.00015 |
| 21 | -0.00028 | -0.00016 | -3.34821 | 0.00002 | 0.00015 |
| 22 | -0.00026 | -0.00014 | -3.39140 | 0.00002 | 0.00013 |
| 23 | -0.00028 | -0.00016 | -3.50789 | 0.00002 | 0.00014 |
| 24 | -0.00026 | -0.00015 | -3.41053 | 0.00002 | 0.00016 |
| 25 | -0.00027 | -0.00016 | -3.23863 | 0.00002 | 0.00020 |
| 26 | -0.00030 | -0.00016 | -3.28711 | 0.00003 | 0.00022 |
| 27 | -0.00028 | -0.00015 | -3.15470 | 0.00002 | 0.00020 |
| 28 | -0.00024 | -0.00016 | -3.34217 | 0.00003 | 0.00023 |
| 29 | -0.00029 | -0.00017 | -3.25763 | 0.00003 | 0.00017 |
| 30 | -0.00025 | -0.00015 | -3.39389 | 0.00002 | 0.00012 |
| 31 | -0.00026 | -0.00015 | -3.44316 | 0.00002 | 0.00014 |
| 32 | -0.00026 | -0.00016 | -3.47160 | 0.00002 | 0.00013 |
| 33 | -0.00026 | -0.00015 | -3.46868 | 0.00002 | 0.00013 |
| 34 | -0.00026 | -0.00014 | -3.18177 | 0.00002 | 0.00012 |
| 35 | -0.00029 | -0.00016 | -3.25680 | 0.00003 | 0.00016 |
| 36 | -0.00028 | -0.00015 | -3.38898 | 0.00002 | 0.00013 |
| 37 | -0.00029 | -0.00016 | -3.18125 | 0.00003 | 0.00018 |
| 38 | -0.00030 | -0.00017 | -3.24647 | 0.00003 | 0.00016 |
| 39 | -0.00026 | -0.00016 | -3.57741 | 0.00002 | 0.00015 |
| 40 | -0.00025 | -0.00015 | -3.15191 | 0.00001 | 0.00018 |
| 41 | -0.00022 | -0.00012 | -2.89202 | 0.00001 | 0.00019 |
| 42 | -0.00026 | -0.00015 | -3.25077 | 0.00002 | 0.00014 |
| 43 | -0.00026 | -0.00017 | -3.42424 | 0.00002 | 0.00014 |
| 44 | -0.00028 | -0.00015 | -3.39530 | 0.00002 | 0.00014 |
| 45 | -0.00026 | -0.00014 | -3.15077 | 0.00002 | 0.00008 |
| 46 | -0.00026 | -0.00014 | -3.09165 | 0.00002 | 0.00011 |
| 47 | -0.00025 | -0.00017 | -3.08000 | 0.00000 | 0.00022 |
| 48 | -0.00030 | -0.00018 | -3.54888 | 0.00003 | 0.00018 |
| 49 | -0.00026 | -0.00018 | -3.68015 | 0.00003 | 0.00021 |
| 50 | -0.00026 | -0.00018 | -3.40624 | 0.00002 | 0.00023 |
| 51 | -0.00025 | -0.00017 | -3.48098 | 0.00003 | 0.00023 |
| 52 | -0.00026 | -0.00018 | -3.48617 | 0.00002 | 0.00024 |
| 53 | -0.00025 | -0.00013 | -3.19307 | 0.00002 | 0.00015 |
| 54 | -0.00027 | -0.00019 | -3.44948 | 0.00001 | 0.00022 |
| 55 | -0.00025 | -0.00013 | -3.19306 | 0.00002 | 0.00015 |

**Supplementary Table S7.** Evapotranspiration elasticities to climatic parameters (Maximum temperature, minimum temperature, wind speed, relative humidity and sunshine duration). Note that the values have not been incorporated in the study. The reasons are discussed in the main manuscript.

Text for **Supplementary table S7**:

Methodology: The method adopted is taken from Yang and Yang^5^, Wang et al.^6^

Data sources: Temperature data was obtained from Climatic Research Unit (CRU) Time-series (TS) data version 4.01 data.^7^

Relative humidity and Wind data was obtained from National Centres for Environmental Prediction (NCEP) and Climate Forecast System Reanalysis (CFSR)^8,9^

Sunshine duration data was obtained from Indian Meteorological Department and World Meteorological Organization (World weather information service).

5. Yang, H. & Yang, D. Derivation of climate elasticity of runoff to assess the effects of climate change on annual runoff. *Water Resour. Res.* **47,** 1–12 (2011).

6. Wang, W. *et al.* The analytical derivation of multiple elasticities of runoff to climate change and catchment characteristics alteration. *J. Hydrol.* **541,** 1042–1056 (2016).

7. Harris, I., Jones, P. D., Osborn, T. J. & Lister, D. H. Updated high-resolution grids of monthly climatic observations - the CRU TS3.10 Dataset. *Int. J. Climatol.* **34,** 623–642 (2014).

8. Dile, Y. T. & Srinivasan, R. Evaluation of CFSR climate data for hydrologic prediction in data-scarce watersheds: an application in the blue nile river basin. *J. Am. water Resour. Assoc.* **50,** (2014).

9. Fuka, D. R. *et al.* Using the Climate Forecast System Reanalysis as weather input data for watershed models. *Hydrol. Process.* **28,** 5613–5623 (2014).

**
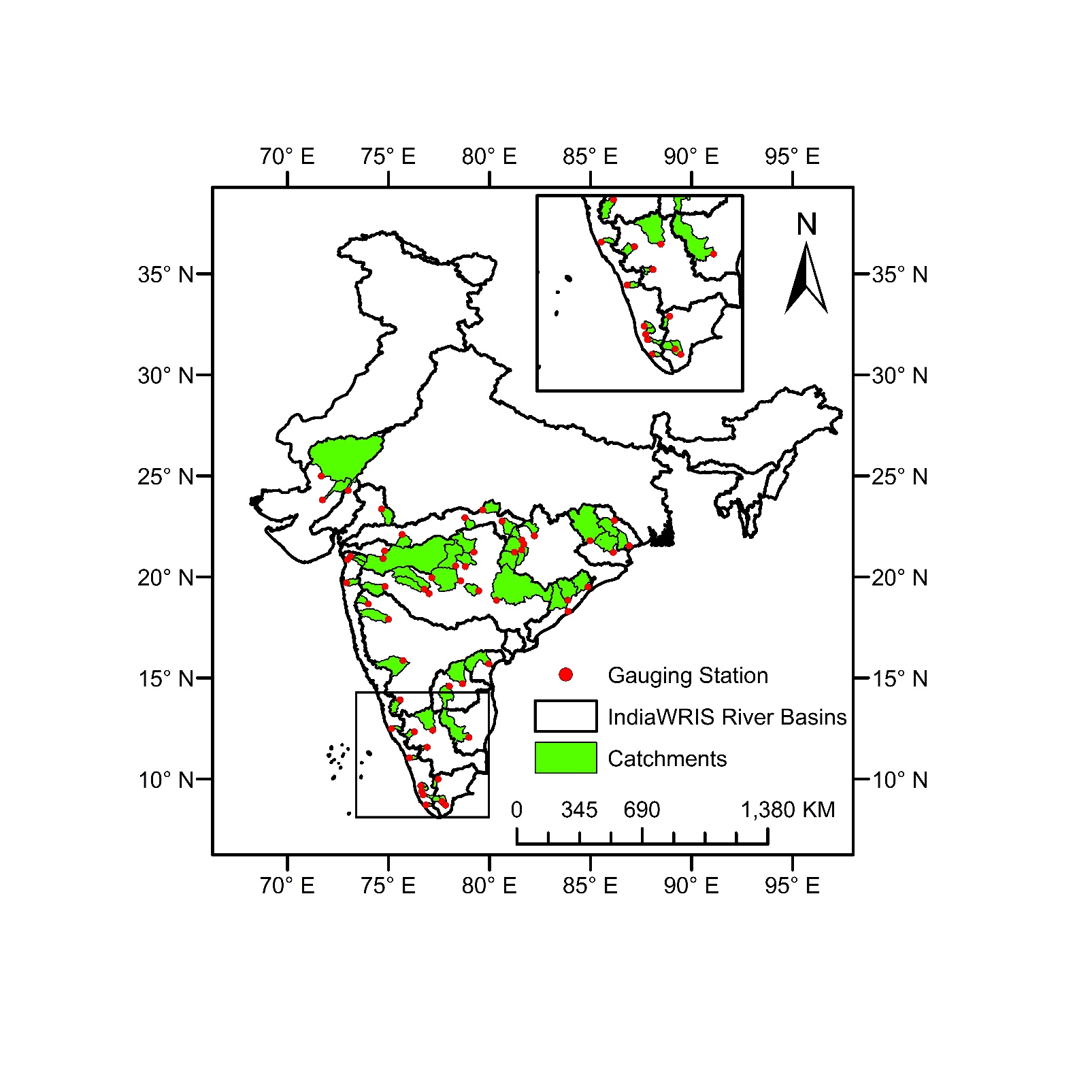
**

**Supplementary Figure S1.** Catchments considered on Peninsular India. The locations of 55 catchments from 17 river basins across Peninsular India with their gauging stations (Shown with red circle) incorporated in the study.

**
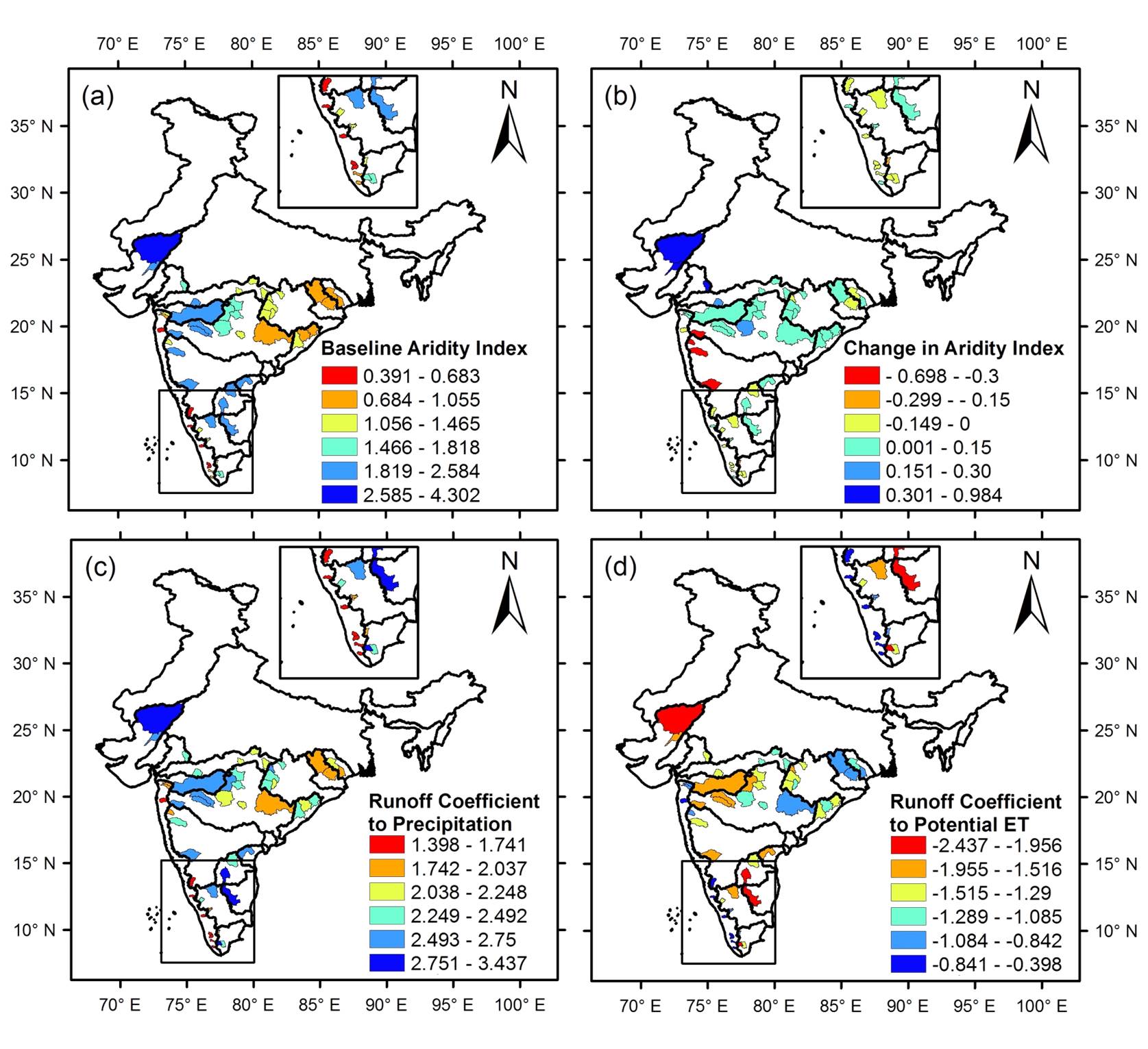
**

**Supplementary Figure S2.** Spatial distribution of aridity indices and runoff coefficients. Distribution of annual average aridity index during (a) baseline period and (b) change in aridity index from baseline to assessment period (c) distribution of runoff coefficient to precipitation and (d) potential evapotranspiration.


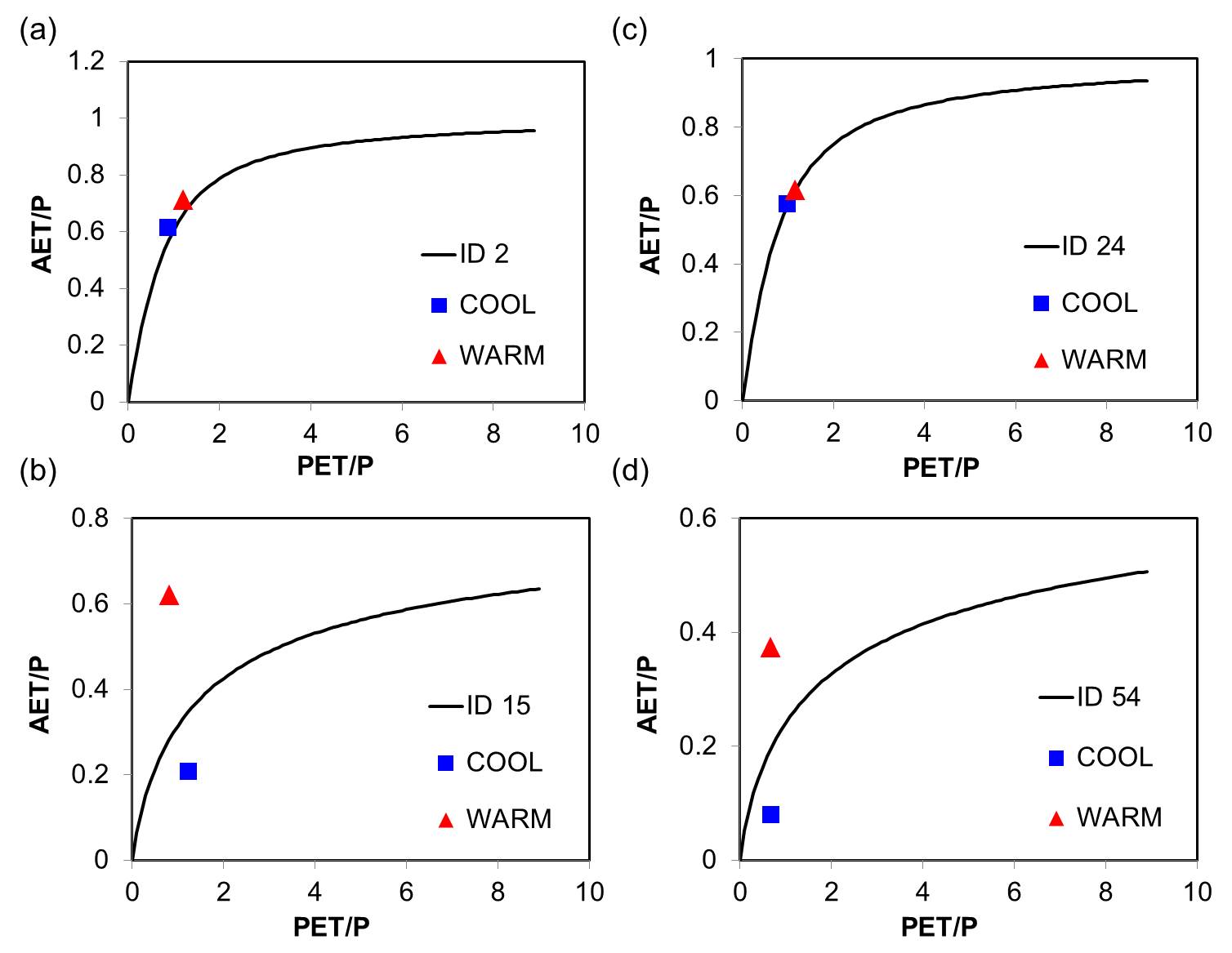


**Supplementary Figure S3.** Representation of catchmnent responses during cool and warm period with theoritical Budyko curve. Responses for two resilient catchments (ID 2 (a) and ID 24 (c)). Responses for two non-resilient catchments (ID 15 (b) and ID 54 (d)). The two resilient catchements have shown the resilience characteristics (Low deviation and high elasticity) unlike the non-resilient catchments.


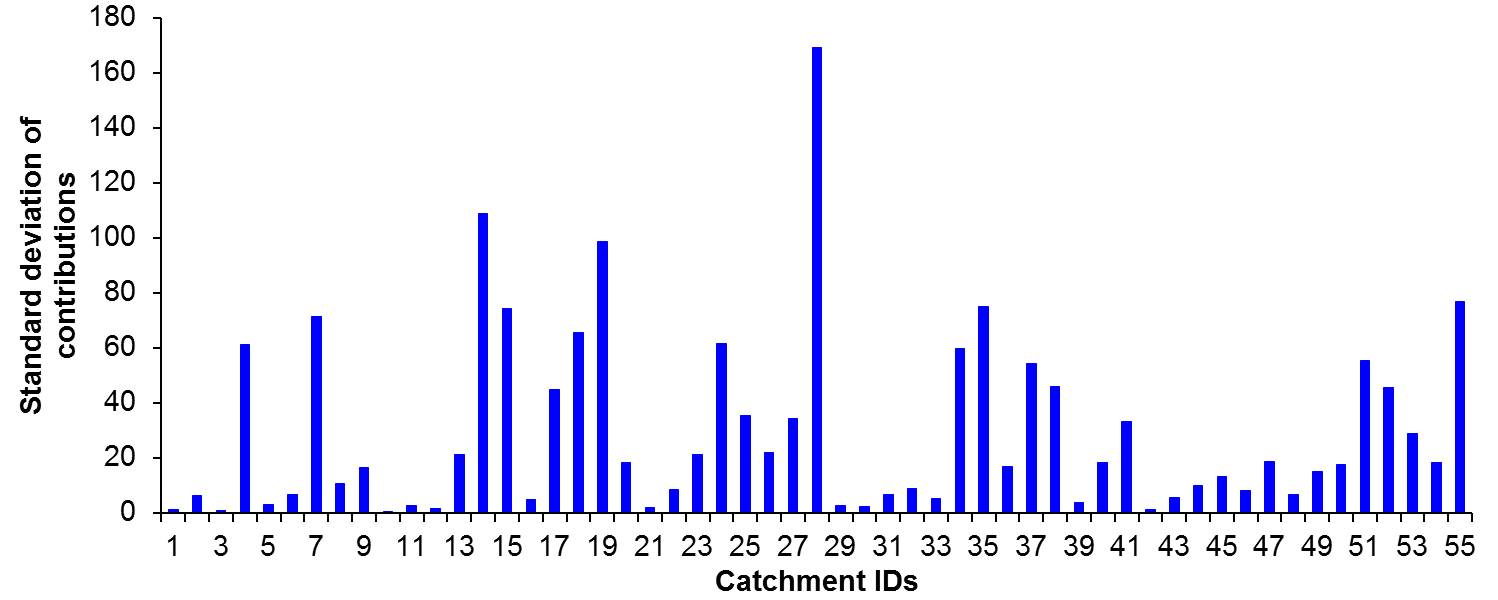


**Supplementary Figure S4.** Dispersion of percentage contributions from climatic variability. Standard deviation of the percentage contributions of climatic variability obtained from eight Budyko based methods for the catchments


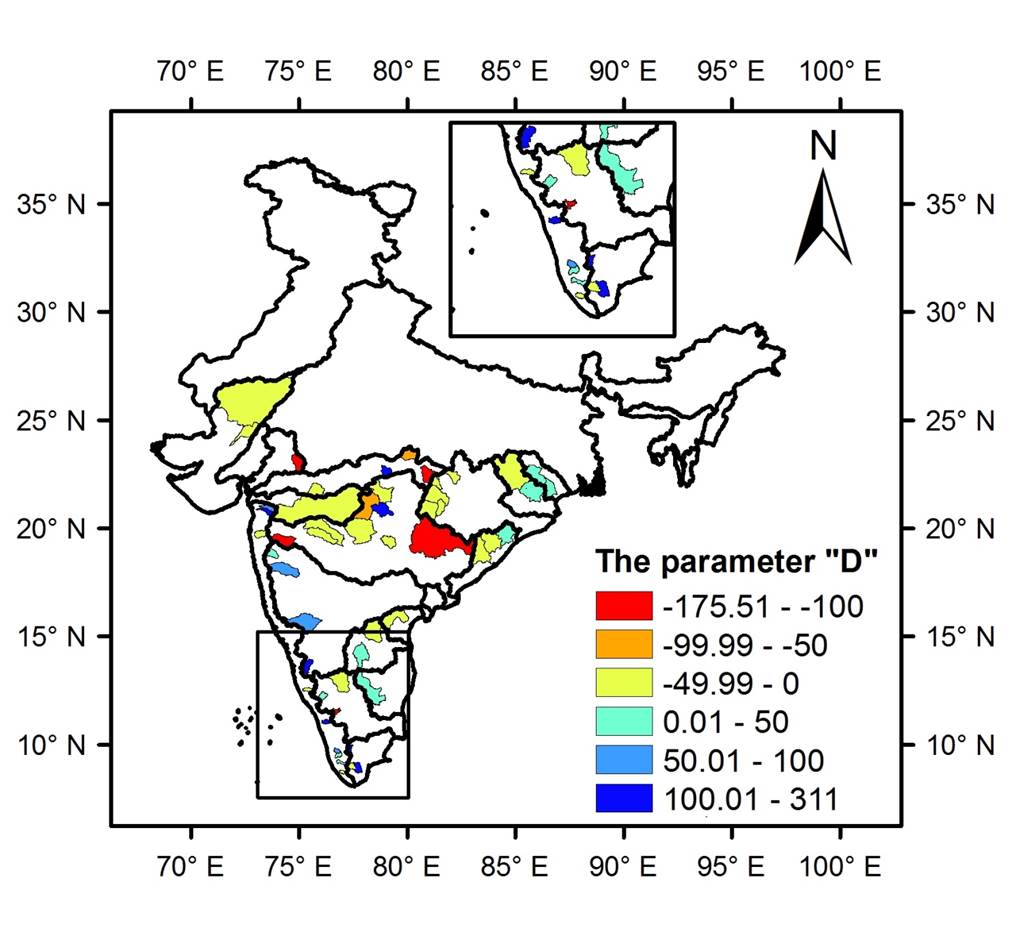


**Supplementary Figure S5.** Spatial distribution of ‘D’ parameter. Spatial distribution of difference between mean of percentage contributions from parametric and non-parametric equations.
